# Advancements in Magnetorheological Foams: Composition, Fabrication, AI-Driven Enhancements and Emerging Applications

**DOI:** 10.3390/polym17141898

**Published:** 2025-07-09

**Authors:** Hesamodin Khodaverdi, Ramin Sedaghati

**Affiliations:** Department of Mechanical, Industrial and Aerospace Engineering, Concordia University, Montreal, QC H3G 1M8, Canada

**Keywords:** magnetorheological foam, multifunctional composites, smart materials, machine learning for material design, fabrication methods, AI-driven enhancements, emerging applications, MR materials

## Abstract

Magnetorheological (MR) foams represent a class of smart materials with unique tunable viscoelastic properties when subjected to external magnetic fields. Combining porous structures with embedded magnetic particles, these materials address challenges such as leakage and sedimentation, typically encountered in conventional MR fluids while offering advantages like lightweight design, acoustic absorption, high energy harvesting capability, and tailored mechanical responses. Despite their potential, challenges such as non-uniform particle dispersion, limited durability under cyclic loads, and suboptimal magneto-mechanical coupling continue to hinder their broader adoption. This review systematically addresses these issues by evaluating the synthesis methods (ex situ vs. in situ), microstructural design strategies, and the role of magnetic particle alignment under varying curing conditions. Special attention is given to the influence of material composition—including matrix types, magnetic fillers, and additives—on the mechanical and magnetorheological behaviors. While the primary focus of this review is on MR foams, relevant studies on MR elastomers, which share fundamental principles, are also considered to provide a broader context. Recent advancements are also discussed, including the growing use of artificial intelligence (AI) to predict the rheological and magneto-mechanical behavior of MR materials, model complex device responses, and optimize material composition and processing conditions. AI applications in MR systems range from estimating shear stress, viscosity, and storage/loss moduli to analyzing nonlinear hysteresis, magnetostriction, and mixed-mode loading behavior. These data-driven approaches offer powerful new capabilities for material design and performance optimization, helping overcome long-standing limitations in conventional modeling techniques. Despite significant progress in MR foams, several challenges remain to be addressed, including achieving uniform particle dispersion, enhancing viscoelastic performance (storage modulus and MR effect), and improving durability under cyclic loading. Addressing these issues is essential for unlocking the full potential of MR foams in demanding applications where consistent performance, mechanical reliability, and long-term stability are crucial for safety, effectiveness, and operational longevity. By bridging experimental methods, theoretical modeling, and AI-driven design, this work identifies pathways toward enhancing the functionality and reliability of MR foams for applications in vibration damping, energy harvesting, biomedical devices, and soft robotics.

## 1. Introduction

Magnetorheological (MR) materials, a class of smart materials, exhibit a rapid, continuous, and reversible change in their viscoelastic properties (including shear modulus, storage, and loss modulus, damping properties, dynamic yield stress, etc.) in response to an external magnetic field. A key advantage of MR materials over other smart materials is their low-power requirement and millisecond-order response time [1]. These materials typically consist of micron-sized, magnetically permeable particles dispersed within a matrix, with various additives often incorporated to enhance specific properties. MR materials can be classified based on several criteria, including the physical state of the matrix (MR fluids and MR solids [2]), the fabrication method (ex situ and in situ [3]), and the type of matrix. MR fluids, the first type of MR material developed, utilize fluids such as silicone oil [4], mineral oil, synthetic oil, or water-based solutions as the matrix [5]. While MR fluids have found numerous applications in damping systems [6], vibration control systems [7], actuation systems [8], medical field [9], and robotics [10], they are susceptible to leakage and particle sedimentation. Strategies such as surface treatment of the magnetic particles [11], the addition of various additives (e.g., surfactants, stabilizers, and thixotropic agents), and the use of higher-viscosity matrices have been explored to mitigate these limitations. This led to the development of MR elastomers, which address the sedimentation and leakage issues associated with MR fluids.

Building upon the development of MR elastomers, other types of MR solids emerged, including MR gels and MR foams. MR solids offer additional advantages, such as the ability to fabricate regular and controllable shapes for diverse applications [2,3]. In recent years, MR foams have garnered significant attention due to their unique properties. Early work on MR foams was conducted by Carlson [12] in 1999, who developed an absorbent, sponge-like material saturated with an MR fluid carrier, introducing the initial concept of an MR fluid foam. Early MR foams typically consisted of MR fluids combined with various types of foam matrices. However, Gong et al. [13] later developed a novel MR foam by embedding carbonyl iron particles (CIPs) within a polyurethane (PU) foam matrix using an in situ polymerization technique. These newer MR foams possess a unique porous and cellular structure, which is formed during the foaming process as gases are chemically generated and trapped within the liquid polymer matrix prior to or during polymerization.

Beyond addressing the leakage and sedimentation issues associated with MR fluids, MR foams offer several distinct advantages over other MR materials due to their unique porous structure. Compared with other MR materials, MR foams have lower density [14], which makes them attractive for specialized applications, such as in the aerospace industry [13]. MR foams also typically show higher MR effect due to their foam’s cellular structure [13], which enables a wide range of achievable properties. Moreover, MR foams exhibit superior vibration and acoustic absorption capabilities, attributed to their gas-filled pores. When exposed to external mechanical or acoustic stimuli, the gas-filled pores contribute to energy dissipation through multiple mechanisms [15]. First, as sound waves or vibrations propagate through the foam, interactions between the moving air and the internal pore walls cause frictional losses, converting part of the energy into heat. Second, the compression and rarefaction of air trapped within the pores lead to additional energy loss through viscous and inertial effects. Finally, the flexible pore walls themselves can undergo micro-resonance, further converting acoustic or mechanical energy into heat and internal deformation. Unlike conventional foams, which rely on passive absorption, MR foams enable active control of vibration and sound [16]. Finally, MR foams can withstand large strains, a property that can be exploited in applications like energy harvesting. In this context, mechanical stress induces a change in magnetic properties, leading to a voltage across a pick-up coil and subsequent energy generation. This application will be discussed in greater detail in the applications section.

The MR effect in MR foams arises from the interaction of the embedded magnetic particles. When subjected to an external magnetic field, these particles become magnetized and align along the field’s direction. The resulting field-induced magnetic forces between the particles enhance the material’s resistance to deformation, leading to a change in shear modulus and other properties [13]. MR foams offer tunable mechanical properties under the influence of magnetic fields, making them well-suited for integration into smart systems and adaptive structures. However, their development presents several challenges. These include achieving uniform particle dispersion within the matrix, balancing magnetic responsiveness with mechanical stability, and ensuring durability under cyclic loading. Despite these challenges, the field continues to advance, driven by the potential of MR foams as adaptive materials in diverse applications, such as vibration control, robotics, and biomedical devices. Ongoing progress in fabrication techniques and the increasing emphasis on sustainable solutions are continually expanding the capabilities of MR foam technology. Furthermore, the MR effect provides a unique platform for investigating particle–matrix interactions under external stimuli, bridging the disciplines of materials science, magnetics, and fluid mechanics, and ultimately facilitating the design of next-generation smart materials.

This review provides a comprehensive and systematic analysis of MR foams, focusing on their development from the perspectives of materials science and mechanical engineering. Unlike prior reviews that primarily emphasize MR fluids and MREs, this work offers a focused exploration of MR foams—an emerging and comparatively under-represented subclass of MR solids. It addresses critical gaps by examining the structure–property relationships, fabrication methods, and magneto-mechanical behaviors that define MR foams, with particular attention to their tunable responses under external magnetic fields. In addition, the review underscores the emerging importance of AI for modeling, property prediction, and design optimization, highlighting how data-driven approaches are increasingly shaping MR foam research. By consolidating developments across synthesis methods, theoretical frameworks, and application-driven performance studies, this paper lays the foundation for understanding current trends and identifying future research directions in the field.

To aid the reader in navigating this comprehensive review, the manuscript is organized into clearly structured sections. Following the introduction, Section 2 explores the fundamental magnetorheological and mechanical properties of MR foams, which form the basis for their unique field-responsive behavior. Section 3 reviews the various fabrication techniques, while Section 4 delves into the material composition and microstructural aspects that govern performance. Section 5 presents theoretical and computational modeling approaches, including both well-established frameworks and promising new methods under current investigation. Section 6 highlights the integration of AI into MR foam research, focusing on property prediction and design optimization. Section 7 provides an overview of the influence of different factors on MR foam performance, and Section 8 outlines their emerging applications. Finally, Section 9 discusses the key challenges, research gaps, and future directions. While these challenges are presented at the conclusion of the review, this placement reflects their inherently multidisciplinary nature—addressing them requires a holistic understanding of MR foam behavior, material composition, processing methods, and application demands, which are established throughout the preceding sections.

## 2. Magnetorheological and Mechanical Properties of MR Foams

MR foams are multifunctional materials that exhibit field-dependent mechanical and rheological properties due to the inclusion of magnetic particles within a porous polymer matrix. Their ability to reversibly alter stiffness, damping, and deformation characteristics under an applied magnetic field makes them highly attractive for different applications. This section delves into the core magnetorheological effect exhibited by these porous composites, which forms the basis of their functionality. Furthermore, it explores the crucial mechanical characteristics of MR foams, including their magnetostrictive strain capabilities and their complex viscoelastic behavior under varying strain amplitudes, encompassing the linear viscoelastic region and the nonlinear Payne effect. Understanding these fundamental properties is essential for tailoring MR foams for diverse applications, ranging from vibration damping and tunable stiffness to soft robotics and actuators.

### 2.1. MR Effect

The MR effect is the defining characteristic that distinguishes MR materials from conventional and other smart materials. This effect is characterized by a reversible change in the viscoelastic properties (including shear yield stress, storage modulus, loss modulus, etc.) of the MR materials when subjected to an external magnetic field. The magnitude and nature of these changes dictate the applicability of the MR materials. The MR effect can be explained by the tendency of the magnetic particles embedded within the carrier medium to reorganize and align parallel to the applied magnetic field. The manifestation and expression of the MR effect vary across different types of MR materials. In MR fluids, it is typically expressed in terms of yield shear stress and apparent viscosity. In MR devices, it is often quantified as a damping force or torque. For MR elastomers (MRE) and MR foams, the storage shear modulus (*G*′) and loss shear modulus (*G*″) are commonly used metrics.

To characterize the linear viscoelastic behavior of MR materials, the material is typically subjected to a sinusoidal deformation while measuring the resulting stress, either in the presence or absence of a varying magnetic field. This process reveals a viscoelastic response with a phase lag (*δ*) between the input strain and the output stress. For instance, the strain and stress in an MR material under sinusoidal normal strain can be expressed as:

(1) $$\varepsilon =\;\varepsilon_{0}\sin\left(\omega t\right)$$

(2) $$\sigma =\sigma_{0}\cos\left(\omega t+\delta \right)$$

where *ω* represents the frequency of the oscillatory strain, and *δ* represents the phase angle or phase lag between strain and stress. From these expressions, we can define two moduli:

(3) $$E^{\prime }=\frac{\sigma_{0}}{\varepsilon_{0}}\cos\left(\delta \right)$$

(4) $$E^{\prime \prime }=\frac{\sigma_{0}}{\varepsilon_{0}}\sin\left(\delta \right)$$

where *E*′ is the tensile storage modulus and *E*″ is the tensile loss modulus. Alternatively, using complex variables, the complex tensile modulus (E*) can be expressed as:

(5) $$\varepsilon =\varepsilon_{0}e^{i\omega t}$$

(6) $$\sigma =\sigma_{0}e^{i\left(\omega t+\delta \right)}$$

(7) $$E^{*}=\frac{\sigma }{\varepsilon }=\frac{\sigma_{0}}{\varepsilon_{0}}e^{i\delta }=\frac{\sigma_{0}}{\varepsilon_{0}}\left(\cos\delta +i\sin\delta \right)=E^{\prime }+iE^{\prime \prime }$$

Following a similar approach and using sinusoidal shear strain, we can derive the shear modulus. The complex shear modulus G* is defined as G* = *G*′ + *iG*″, where *G*′ is the shear storage modulus and *G*″ is the shear loss modulus. The storage modulus represents the stored energy (the elastic component), while the loss modulus represents the energy dissipated during deformation [17].

The loss tangent (*tan δ*) is also defined as:

(8) $$\tan\delta =\frac{\mathrm{Energy}\;\mathrm{Loss}}{\mathrm{Energy}\;\mathrm{Stored}}=\frac{G^{\prime \prime }}{G^{\prime }}=\frac{E^{\prime \prime }}{E^{\prime }}$$

which is commonly used in MR materials, indicating the energy dissipation during deformation.

The MR effect in MR materials is often characterized in terms of relative and absolute MR effects. The relative MR effect is defined as Δ*G*′/*G*_0_′, where *G*_0_′ is the zero-field storage modulus and Δ*G*′ is the field-induced increment in the storage modulus. The absolute MR effect is simply Δ*G*′. Similar relationships hold for the loss modulus:

(9) $$G^{\prime }\left(H\right)=G_{0}^{\prime }+\Delta G^{\prime }\left(H\right)$$

(10) $$G^{\prime \prime }\left(H\right)=G_{0}^{\prime \prime }+\Delta G^{\prime \prime }\left(H\right)$$

where $G_{0}^{\prime \prime }$ represents the zero-field shear loss modulus and $\Delta G^{\prime \prime }\left(H\right)$ represents the changes in the shear loss modulus due to the application of a magnetic field $\left(H\right)$.

The MR effect in view of damping is typically described by the change in loss modulus (*G*″) or the loss factor (*tan δ*) under magnetic field activation. According to Yang et al. [18], there are three types of damping mechanisms in MR solids: intrinsic damping (*D_C_*), interfacial damping (*D_I_*), and magneto-mechanical damping (*D_M_*). The total damping capacity of an MR solid can, therefore, be expressed as: *D_MRE_* = *D_C_* + *D_I_* + *D_M_*. Since MR solids are particle-reinforced composites made from two distinct materials, the rule of mixtures can be applied to describe the intrinsic damping. The overall intrinsic damping depends on the content and damping characteristics of each component. However, the damping capacity of magnetic particles (such as CIPs) is much lower than that of the polymer matrix, making their contribution to intrinsic damping negligible. The interfacial condition also plays a significant role in determining the damping behavior of MR solids. Based on Yang et al. [18], interfacial bonding can be categorized into three types: ideal, strongly bonded, and weakly bonded interfaces. Ideal interfaces transfer stress and strain but do not contribute to damping. In strongly bonded interfaces, damping arises from stress concentration near the particle surfaces. In contrast, weakly bonded interfaces exhibit damping due to internal friction from relative motion between the matrix and particles. Strong interfacial bonding generally occurs at low particle content and small strain amplitudes. Finally, magneto-mechanical damping is mainly governed by magneto-mechanical hysteresis. Since most MR-based devices operate under vibration, investigating this hysteresis-based damping mechanism is essential for performance optimization [18].

Stiffness is one of the key mechanical properties of MR solids, which can be actively tuned in response to an external magnetic field. Barron III et al. [19] investigated stiffness tuning in MREs and proposed a new hybrid composite architecture that incorporates both rigid magnetic particles and MR fluid droplets within an elastomeric matrix. They developed a predictive model to describe stiffness tuning by combining the zero-field composite modulus *E*_*c*,*B*=0_ and a magnetic contribution *E_mag_*, assuming additive behavior similar to a parallel spring arrangement. This leads to the expression *E_c_* = *E*_*c*,*B*=0_ *+ E_mag_*. To evaluate tunability, they normalized this relationship and defined the MR enhancement factor as *E_c_*/*E*_*c*,*B*=0_, where larger values indicate greater tunability. When this factor approaches 1, the influence of the magnetic field becomes negligible, indicating a physical limit to tunability, especially when the zero-field modulus is already high. They also defined the MR effect in terms of stiffness as (*E_mag_*/*E*_*c*,*B*=0_) × 100, representing the relative increase in modulus due to magnetic activation. This model was validated across different composite structures, demonstrating its effectiveness in guiding the design of MREs for applications requiring extreme and rapid stiffness modulation.

### 2.2. Magnetostrictive Strain

In addition to field-dependent viscoelastic properties, MR foams exhibit notable actuation capabilities due to their inherent magnetostrictive behavior. Magnetostrictive strain in MR foams refers to the fractional change in the dimensions of an MR foam in response to an applied magnetic field, specifically along the field’s direction. Several types of magnetostrictive strain are commonly defined. Longitudinal magnetostriction refers to the change in length of a material in the direction parallel to the applied magnetic field ($∆l/l_{o}$). This magnetostriction can be either positive (foam elongation) or negative (foam contraction), and it has been extensively investigated through experimental [20,21], analytical [22], and numerical [23] approaches. Magnetostriction can be attributed to the induced dipole–dipole interactions among the magnetic particles within the polymer matrix [24]. When an external magnetic field is applied, the initially non-magnetized particles within the polymer matrix become magnetized, inducing dipole moments that cause them to rotate and align along the field direction. As alignment increases, these particles exert forces on the surrounding matrix, leading to macroscopic deformation in the direction of the field. Depending on their relative orientation, the magnetized particles experience attractive or repulsive dipole–dipole interactions, which can promote the formation of chain-like structures aligned with the field. This structural reorganization amplifies the magnetostrictive response by imposing directional stress on the matrix, with the overall effect influenced by factors such as particle concentration, matrix stiffness, and field strength [20].

In 2008, Bednarek [25] fabricated an MR foam using elastic silicone rubber as the matrix and pure iron particles with sizes ranging from 0.10 to 0.15 mm as the filler. This foam exhibited a high longitudinal magnetostriction strain percentage for magnetic fields exceeding 1 T. The porous structure of the matrix was achieved through a method of rinsing out soluble NaCl salt. The fabricated MR foam demonstrated a 3.95% longitudinal magnetostriction under a 7 T magnetic field, with a porosity factor of 0.15 (defined as the ratio of the total pore volume in the non-deformed composite to the volume of the non-deformed composite) and a filling factor of 0.45 (defined as the ratio of the total ferromagnetic particle volume to the volume of the non-deformed composite).

Considering the above paper, which utilized magnetic fields ranging from 1 T up to 7 T, it is worth noting that in practical applications, the magnetic field strengths used in MR devices typically range from 50 to 500 mT, depending on the actuator or damping system design [26]. For example, commercial MR dampers and isolators commonly operate under fields of 100–600 mT, achievable using compact permanent magnets or small electromagnets [27,28]. In contrast, field strengths exceeding 1 T and especially those approaching 7 T are generally confined to laboratory settings, where they are used to explore the upper limits of MR material response. These high-field experiments are valuable for understanding saturation behavior and maximum achievable stiffness; however, they are not representative of most real-world use cases due to the substantial power requirements and bulky hardware needed to generate such fields.

Later, Guan et al. [20] investigated the magnetostrictive effect of MREs composed of CIPs dispersed within a silicone rubber matrix. Their results indicated that the longitudinal magnetostrictive effect increased from 0.0025% to 0.0132% with an increase in CIP volume fraction from 15 wt.% to 27 wt.% under a 0.8 T magnetic field. Furthermore, as the magnetic field intensity approached 0.5 T, the increase in magnetostriction slowed, suggesting a tendency toward saturation. They also reported that the orientation of the particles significantly influenced the magnetostrictive performance; samples with longitudinally aligned particles showed 2.3 times greater longitudinal magnetostriction than samples with transversely aligned particles.

Wang et al. [29] fabricated an MR foam with a cellular structure using natural rubber latex and CIPs as the main ingredients, with N, N′-dinitroso pentamethylene tetramine as the blowing agent. Curing and foaming were performed in an air-circulating oven for 90 min at 140 °C. The resulting MR foam exhibited excellent magnetostriction performance. Specifically, the positive longitudinal magnetostriction value of MR foam with cell sizes of 0.48 mm and 0.37 mm was approximately 11.0% and 9.2%, respectively, under a magnetic field of 1.0 T. This was significantly larger than that (4.6%) of a non-porous MR bulk (MR elastomer) sample, indicating a larger magnetostriction value for the MR foam with the larger cell size at a similar porosity. Furthermore, when the magnetic field was completely switched off, all samples showed almost no remanent magnetostriction and negligible magnetostriction hysteresis. In addition, the MR foam also demonstrated high magnetic sensitivity in magnetostriction, achieving a substantial magnetostriction of 5.1% at a low magnetic field of 0.2 T, which corresponds to a magnetic sensitivity of approximately 2.55 × 10^−2^%/mT.

Rohim et al. [30] developed AI-driven models to predict the magnetostriction behavior (strain and normal force) of MR foams with varying CIP compositions. These models effectively captured the nonlinear relationships between CIP composition, magnetic field, strain, and normal force. Based on the root-mean-square error and R-squared values, the AI-driven models demonstrated high accuracy in predicting magnetostriction behavior. A more detailed study of these models is presented in the subsequent sections.

### 2.3. Linear Viscoelastic Region, Payne Effect, and Strain-Dependent Behavior

The linear viscoelastic (LVE) region of MR solids refers to the range of strain within which the material exhibits a linear relationship between stress and strain, defining the shear strain limit before permanent structural deformation occurs [31]. Identifying the LVE limit is essential to preventing sample damage during testing and application. This limit is determined at the point where *G*′ (storage modulus) and *G*″ (loss modulus) become dependent on the strain amplitude [32]. Wereley et al. [33] defined the LVE limit for MR foams as the point where the storage modulus deviates by 10% from its plateau value. Agirre-Olabide et al. [32] later proposed using the loss factor to determine the LVE region in MREs, as it provides a more restrictive limit than the storage modulus. Beyond the LVE region, the Payne effect becomes significant. This effect describes the nonlinear viscoelastic behavior of MR materials under large strain amplitudes. The Payne effect is characterized by a decrease in the storage modulus with increasing strain amplitude. This phenomenon is attributed to the non-recoverable alterations in the material’s microstructure, such as the disruption of particle–particle interactions or the breakdown of network-like structures formed by magnetic particles under the influence of a magnetic field [34]. Increased strain leads to larger interparticle distances, weakening the interaction forces between magnetized particles, resulting in material softening and a reduction in the modulus.

## 3. Fabrication Techniques

MR foams can be synthesized using two primary fabrication methods: ex situ and in-situ. The ex situ method involves soaking a pre-formed foam structure in an MR fluid mixture, which typically consists of magnetic particles dispersed in a liquid medium such as a fluid [35,36], elastomer, or plastomer [37]. After the soaking process, the foam is squeezed to remove the excess fluid mixture. The foam, now coated internally with the MR mixture, is then left to dry (or cure) for several hours [38]. In contrast, the in situ method integrates magnetic particles directly into the foam matrix during fabrication. This approach ensures a more uniform particle distribution and stronger adhesion within the material. In the following, ex situ and in situ methods are discussed.

### 3.1. Ex Situ Method

To date, two primary types of foam matrix have been used for MR materials fabricated using the ex situ method: metal foams [39,40] and polymer foams. Applications of metal foam-based MR materials remain relatively rare. While metal foams offer advantages such as high stiffness, fire resistance, low moisture absorption, and recyclability, their widespread commercial adoption is limited by manufacturing challenges and the high cost associated with producing the porous, absorbent metal foam matrix.

Polymer foams are commonly used as porous, absorbent matrices due to their ease of preparation and excellent mechanical performance [41], ability to provide good bending properties [42], excellent viscoelastic properties [43] and excellent sound insulation [44]. These polymeric foams consist of a polymer matrix with embedded bubbles, which can be either open-celled or closed-celled, formed from thermoset or thermoplastic polymers [45,46].

In ex situ fabrication methods, the density of the foam plays a crucial role in determining the final properties of the fabricated MR foam. The foam’s structure influences its fluid absorption capacity; lower density and the presence of chain extenders generally lead to greater fluid mixture absorption. However, using excessively low-density foams can result in the rupture of finer cells and increased fluid mixture absorption, potentially leading to polymer chain failure. Therefore, selecting an appropriate polymer foam with the desired density and pore size is essential for successful fluid incorporation [47]. Fluid mixture absorption is primarily influenced by the foam’s density and is less dependent on the viscosity and temperature of the fluid mixture. This relative insensitivity to temperature allows foams to maintain a consistent fluid mixture absorption rate under typical environmental conditions. Furthermore, the foam’s density can be tailored to achieve the desired level of fluid mixture absorption, making it a versatile material for various applications [48].

While the ex situ fabrication method can achieve a substantial MR effect, as demonstrated by Ge et al. [37] (who observed an 820% MR effect by fabricating MR foam with 40, 60, 70, and 80 wt.% CIP after dipping highly porous foam into a PU and CIP mixture and allowing it to rest for 72 h), this approach has several drawbacks. First, the ex situ method requires an additional manufacturing step to transform a pre-existing foam into an MR foam. Second, the magnetic particles tend to agglomerate during the soaking process, adhering to the foam’s pores rather than being embedded within the struts. This can reduce the MR foam’s efficiency [49]. Agglomeration leads to non-uniformity, where some regions exhibit high stiffness and magnetization while others remain largely inactive. As a result, the foam demonstrates inconsistent magneto-mechanical responses and reduced long-term durability, particularly under cyclic loading or mechanical stress. To mitigate these issues, two effective strategies have gained increasing attention. The first is particle surface treatment or coating, such as silane coupling agents or polymer shells, which enhance particle–matrix compatibility, reduce surface energy, and help prevent aggregation [50]. The second is particle size control, as studies have revealed a strong correlation between particle morphology and size, indicating the existence of an optimal particle size for maximizing performance [51]. The drawback in the ex situ fabrication method is that the foam primarily acts as a support structure rather than the primary matrix. This limits the performance of the ex situ method because the magnetic particles are attached to the pore surfaces rather than being integrated into the struts or pore boundaries. Consequently, the magnetic particles can be easily dislodged from the foam structure, restricting the long-term applicability of the fabricated MR foams [52].

### 3.2. In Situ Method

**Figure 1:**
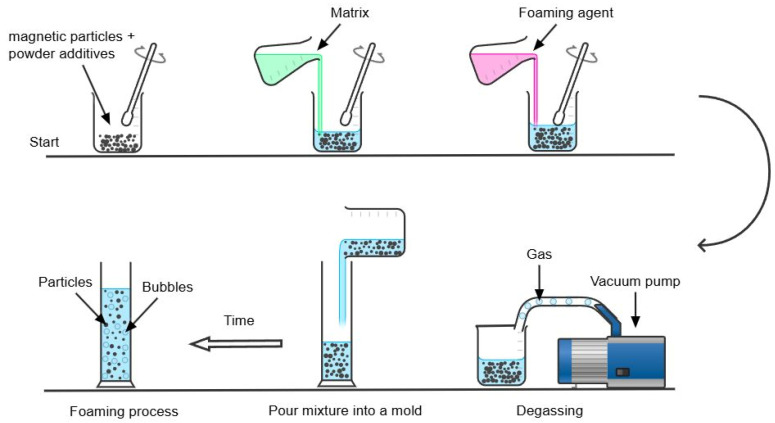
The general synthesis process of MR foam (isotropic).

**Figure 2:**
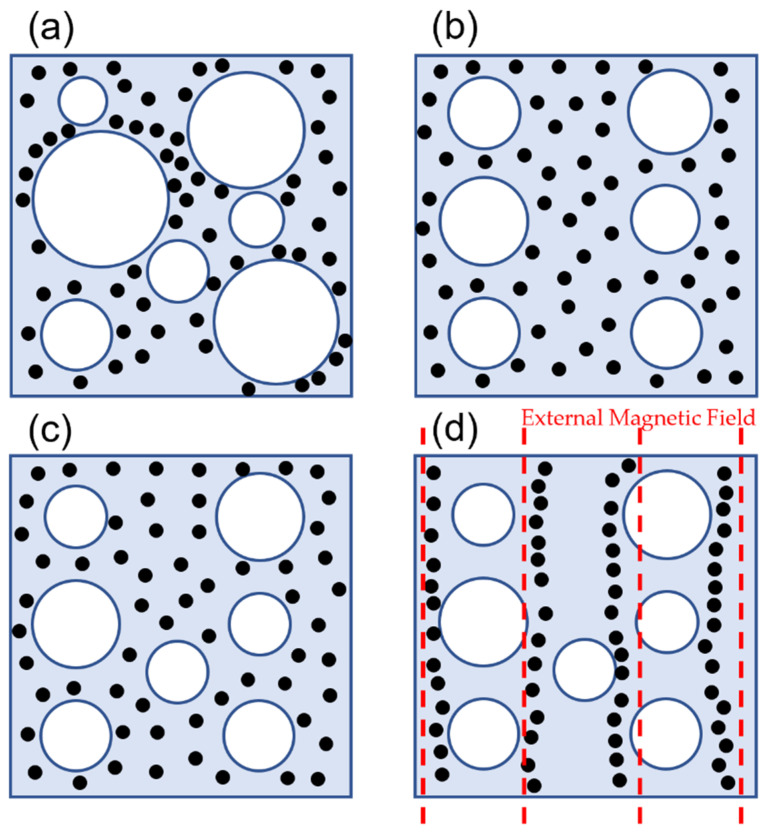
Illustrations of (**a**) non-uniform and (**b**) uniform magnetic particle distributions; (**b**) enhanced uniformity achieved through constrained foaming [53,54]; (**c**) isotropic and (**d**) anisotropic magnetic particle arrangements under the influence of an external magnetic field. (Solid circles represent magnetic particles, hollow circles represent bubbles, and red dashed lines indicate external magnetic field lines.).

To address the limitations of the ex situ fabrication method, the in situ method has been developed to offer improvements in the stability of the particle–matrix interface, wettability, and reduced particle agglomeration within the matrix. The in situ method is generally considered a simpler synthesis process for MR foams due to, often, fewer separate steps and its more straightforward nature, which can potentially reduce overall fabrication time and specialized equipment requirements. However, it is important to note that this practical simplicity does not necessarily correlate with superior material performance, especially given the potential challenges in achieving uniform particle dispersion and the collapse of the MR foam morphology. In this method, the chemical precursors of the foam are mixed directly with the magnetic particles at the beginning of the preparation stage. The mixture then undergoes a foaming process, resulting in a foam structure with the magnetic particles embedded throughout. In situ fabrication methods aim for the magnetic particles to be embedded within the foam’s struts, becoming an integral component of the MR foam’s solid matrix, rather than merely adhering to pore surfaces as in ex situ approaches. However, it is important to note that the uniformity of particle dispersion within these struts can still vary depending on specific processing conditions. In situ fabrication methods are generally preferred due to the improved particle distribution and adhesion. Figure 1 summarizes the typical in situ synthesis process for isotropic MR foam samples. However, challenges such as non-uniform particle dispersion and potential collapse of the MR foam morphology during processing have been observed and remain a risk in production [53,54]. A schematic view of non-uniform particle dispersion in the in situ fabrication method and the effect of constrained foaming in promoting more uniform particle dispersion are shown in Figure 2. This technique will be discussed in more detail in Section 7.

Rohim et al. [30] described an in situ MR foam fabrication process as follows: First, CIPs (diameter 3–5 µm) were suspended in PU polyol and stirred mechanically for 20 s at a constant speed of 550 rpm. Second, diisocyanate was added to the mixture, followed by another stirring for 20 s at a constant speed of 550 rpm. Finally, the resulting mixture was poured into a cylindrical mold to initiate the foaming process. After foaming was complete, the MR foam sample was allowed to cure for 24 h at room temperature before characterization.

### 3.3. Influence of Curing Conditions (Isotropic vs. Anisotropic)

The fabrication of MR materials involves two primary curing conditions that significantly influence the material’s properties and behavior: isotropic and anisotropic. Anisotropic curing is characterized by the alignment of magnetic particles during fabrication under the influence of an external magnetic field [55,56]. Conversely, isotropic curing involves a random distribution of magnetic particles during fabrication in the absence of an external magnetic field [57]. A schematic view of isotropic and anisotropic curing and the effect of an external magnetic field during curing on the chain-like particle distribution structure have been shown in Figure 2.

D’Auria et al. [58] fabricated MR foam by incorporating iron and barium particles into the struts of open-cell PU foam structures under a controlled applied magnetic field. They demonstrated that for samples containing 25 wt.% Fe particles (diameter < 44 µm), anisotropic curing conducted under an external magnetic field of less than 0.1 T significantly enhanced their compressive strength. Specifically, the compressive yield stress along the alignment direction increased from 14.3 kPa in isotropic samples to 43.2 kPa in anisotropic samples (50 mm × 20 mm × 10 mm in length × width × thickness), whereas samples with randomly dispersed magnetic particles showed a decrease in compressive stress.

## 4. Material Composition and Microstructural Properties

The performance of MR foams is fundamentally governed by their material composition and resulting microstructural properties. This section investigates the intricate relationship between the constituent materials and the macroscopic behavior of MR foams, exploring how specific choices in matrices, magnetic fillers, and additives influence their magnetic and viscoelastic characteristics. By elucidating these aspects, we aim to provide a comprehensive overview of how material selection and microstructural engineering contribute to the functionality and versatility of MR foams.

### 4.1. Types of Polymeric and Metallic Matrices

As previously discussed, two primary types of matrices are used in MR foam fabrication: metal foams and polymer foams. The metallic matrices employed in MR foam fabrication include nickel (Ni) [59], iron (Fe) and copper (Cu) [60] foams. A variety of polymeric foam matrices have also been used, including polyurethane (PU) foam, poly (ether sulfone) (PES) [61], poly(ethylene-2,6-naphthalate) (PEN) [62], polyimide (PI) foam [63], and hypromellose phthalate (HP-55) [64].

Among polymeric foam matrices, PU foam has garnered significant attention for MR applications due to its favorable balance of mechanical and physical properties. PU foams typically exhibit elongation at break ranging from 150% to 300%, low density between 30–60 kg/m^3^, and high fatigue resistance, making them suitable for repeated deformation in dynamic environments. In contrast, silicone foams generally possess higher densities (~100–300 kg/m^3^) and lower elongation at break (~50–150%), which limit their compliance and increase system weight. Ethylene–vinyl acetate (EVA) foams, while lightweight, often have lower resilience and poorer energy dissipation, with elongation typically below 150% and less favorable damping behavior under cyclic loads. These distinctions are crucial in MR foam applications: the high flexibility and low density of PU enable greater field-induced strain modulation, improved magneto-mechanical tunability, and more efficient particle alignment during fabrication while minimizing added mass in vibration or impact mitigation systems. Therefore, PU-based matrices remain one of the most effective platforms for developing adaptive and lightweight MR foam devices [65].

Furthermore, PU foam offers remarkable versatility in tailoring its properties by adjusting the formulation and modifying the synthesis and processing conditions. PU foam is typically synthesized using two primary components: polyol and isocyanate. In a common formulation, a polyether polyol (PPG)-based triol (density: 1.03 g/mL) serves as one key ingredient, while 4,4′-methylene bis (phenyl isocyanate) (density: 1.00 g/mL) acts as the second reactant. During the foaming process, the chemical reaction between these components generates gas bubbles that become trapped within the mixture, creating the characteristic porous structure of the foam [46,66].

Khaidir et al. [45] described a specific in situ MR foam fabrication process. CIPs, comprising 50 wt.% of the total polyol amount (approximately 10 g), were added to the polyol and stirred mechanically at 550 rpm for approximately 30 s. Subsequently, 50 wt.% isocyanate (10 g) was added to the polyol–CIP mixture and stirred for an additional 20 s. The resulting mixture was immediately poured into an open cylindrical mold, initiating the foaming process and shaping the MR foam. Gong et al. [13] used polyether triol (Type: TMN-3050; hydroxyl value: 56) as the matrix foam. Their process involved ball milling a mixture of polyether triol, CIPs, glycerol, triethylenediamine (catalyst), a foam stabilizer, and water. Dibutyltin dilaurate (DBTDL) catalyst and isocyanate (PAPI, Type: PM-200) were then added to the dispersion, and the mixture was quickly poured into a mold coated with a release agent. After the foaming process, the PU MR foam was further aged in a 70 °C oven for 1 h.

Hu et al. [67] employed an ex situ fabrication method to create a nanowire/PU sponge-enhanced MR elastomer. Their research involved immersing a porous PU/silver nanowire (PU/AgNW) medium into a CIP-doped PDMS matrix. Their findings indicated good sensitivity properties of the resulting MR elastomer foam under cyclic tensile and compressive loading, suggesting its potential use in damping and stiffness-tunable devices. Ge et al. [37] utilized a dual-matrix approach, combining a low crosslinking PU polymer with a porous PU sponge to enhance the properties of MR foam for broader technological applications. When the CIP content increases to 80 wt.%, the magnetically induced modulus of the MR foam can reach up to 7.34 MPa, with a corresponding relative MR effect of 820%.

Diguet et al. [68] used a commercially available bi-component PU foam system (part A: isocyanate; part B: polyol) mixed at a 57:100 weight ratio (A:B) and stirred for 30 s. Iron (Fe) particles were initially mixed into component A, followed by the addition of component B and an additional 30 s of stirring. Norhaniza et al. [69] and Rohim et al. [30] also used a commercially available two-part system consisting of (a) phenyl isocyanate (density: 1.00 g/mL) and (b) a PPG-based triol (molecular weight: 6000 g/mol; density: 1.03 g/mL). Muhazeli et al. [70] used a rigid PU foam system composed of a polyether polyol reactant (RG135NFDH1) and polymethylene polyphenyl polyisocyanate (4,4′-diphenylmethane diisocyanate) as the PU matrix components. Yang et al. [71] proposed a one-step method for fabricating 3D microporous MR foam based on a PU matrix. CIPs at various mass ratios were mechanically mixed with polyether polyol and methylene diphenyl diisocyanate (MDI). During the reaction, mechanical stirring incorporated air into the mixture. The crosslinking reaction between the polyether polyol and MDI caused the mixture to expand, forming pores uniformly throughout the material. Next, 3 wt.% dibutyl phthalate (DBP) was added as a plasticizer to soften the PU and slow down the foaming process. After mechanical blending for 5 min at room temperature, the mixture was poured into an aluminum mold for crosslinking.

Ju et al. [72] used ammonium bicarbonate (NH_4_HCO_3_) as a foaming agent to produce MR foam, controlling the porosity by varying the amount of NH_4_HCO_3_, which decomposes into CO_2_ during curing. The manufacturing process involved initially mixing the NH_4_HCO_3_ with silicone rubber and then adding CIPs; all ingredients were stirred for approximately 10 min. The mixture was then placed in a vacuum oven to remove air bubbles and subsequently packed into an aluminum mold. Finally, the mixture was cured at 100 °C for 2 h. During curing, the applied heat decomposed the NH_4_HCO_3_ into NH_3_, CO_2_, and H_2_O, creating the porous structure. Their results showed that a non-porous sample exhibited a higher storage modulus than a porous sample. Also, the increment of NH_4_HCO_3_ content increased the porosity of the sample and decreased the shear storage modulus. They also noted that the loss factor increased with increasing porosity (i.e., increasing NH_4_HCO_3_ content). Wang et al. [29] used natural rubber as the polymer matrix for MR foam. They reported that mixing natural rubber with CIPs resulted in a higher magnetostriction (up to 12% at 1 T) compared to using silicone rubber. They developed a new class of magnetic rubber foam with a cellular structure synthesized via a one-step solution foam processing method. This magnetic rubber foam exhibited a larger magnetostriction (12.0%) than magnetostrictive elastomers, along with low remanent magnetostriction and a rapid response time (1.0 s).

Choi et al. [73] used a commercially available silicone foam, Soma Foama 15 (a soft, two-component platinum silicone flexible foam) as the matrix foam. They considered this material as an adaptive cushioning material designed to protect payloads from a wide range of impact loads. Ling et al. [63] experimentally investigated the properties of polyamide/Fe_3_O_4_ composite foams using an in situ dispersion method. Their findings revealed a strong dependence of magnetic behavior on the size of the nanoparticle filler, which consequently influenced the superparamagnetic behavior of the materials.

### 4.2. Characteristics of Magnetic Fillers

In MR foams, various types of magnetic particles are used as fillers, and their characteristics are crucial in determining the material’s magnetic and viscoelastic performance. Key particle features include physical properties such as size, magnetic softness or hardness, and shape anisotropy, as well as chemical composition, all of which indirectly influence the MR effect.

Ferromagnetic, ferrimagnetic, and paramagnetic materials like maghemite, magnetite ferrite, chromium dioxide, iron, and CIPs have been generally used as magnetic components in MR materials [74]. CIPs are the most widely used magnetic particles in MR foam fabrication, followed by barium (Ba) particles [49]. CIPs are preferred due to their high permeability, low remanent magnetization, and high saturation magnetization. These properties contribute to strong interparticle attraction and a reversible MR effect [75].

The size of magnetic particles in MR foams typically ranges from nanometers to micrometers. While research suggests that the MR effect does not increase monotonically with particle size, MR foams fabricated with micrometer-sized particles offer higher MR effect and advantages in terms of ease of fabrication compared to those using nanometer-sized particles [76].

### 4.3. Additives

Various additives are incorporated into MREs and MR foams to enhance specific properties, such as rheological behavior, structural morphology, thermal stability, and mechanical strength. These additives are strategically used to facilitate the fabrication process and optimize the performance of MR materials in various applications. Among the most commonly used additives in MR solids are plasticizers and softening agents, which improve flexibility, processability, and workability. In MREs, plasticizers function as lubricants, reducing intermolecular adhesion and enabling the rubber matrix’s molecular chains to glide more easily. The most frequently used plasticizers in MREs include mineral oils, phthalate esters, and silicone-based oils [77].

Lokander et al. [78] investigated the effect of mineral-oil plasticizers on MREs, reporting that the incorporation of these plasticizers led to a significant increase in shear storage modulus. Their findings indicated that the absolute MR effect remained independent of the matrix material, with softer matrices exhibiting a greater relative MR effect. Specifically, incorporating hydrocarbon oil into the MR material enhanced the relative MR effect by up to 25%, achieving a storage modulus of 2.5 MPa. Similarly, Chen et al. [79] explored the influence of mineral oil plasticizers on the shear storage modulus of anisotropic MREs based on natural rubber. Their study demonstrated that a plasticizer content of 10 wt.% resulted in an initial storage modulus of 1.4 MPa and an absolute MR effect of 0.23 MPa. Increasing the plasticizer content to 20 wt.% reduced the initial shear storage modulus to 0.90 MPa while increasing the absolute MR effect to 0.7 MPa. Furthermore, the anisotropic alignment of magnetic particles played a significant role in enhancing the MR effect by promoting strong particle interactions and reducing energy dissipation. The presence of plasticizers facilitated faster magnetic particle movement within the matrix compared to non-plasticized samples.

Another important category of additives in MR solids is carbon-based additives, such as graphite [80], carbon nanotubes (CNTs), and carbon black [81]. These additives improve mechanical strength during processing and enhance electrical conductivity. Li et al. [82] reported that the addition of graphite significantly increased the electrical conductivity of MREs, reducing resistance by over 85%. Tian et al. [80] demonstrated that incorporating 23.81% micrometer-sized graphite particles into an MR elastomer increased the storage modulus by up to 1 MPa compared to samples without graphite.

While most studies on additive effects have focused on MR elastomers, research on the influence of additives in MR foams remains limited. Existing studies have primarily investigated the use of magnetic and non-magnetic particles such as silica nanoparticles [45,46,62,83] with fewer efforts directed toward tailoring interfacial chemistry or using functional additives optimized for foam structures. Silica nanoparticles have been shown to enhance the thermal and rheological properties of MR foams by increasing intermolecular interactions. They act as nucleating agents, promoting the formation of silane coupling bonds, which improves particle–matrix adhesion.

A more detailed discussion on the effect of different particles on MR foams is provided in Section 7. However, further studies are needed to explore the impact of other types of additives, such as conductive and thermal enhancers, crosslinking agents, curing additives, dispersants, surfactants, plasticizers, and softening agents, on the structural and magnetorheological behavior of MR foams.

## 5. Methods to Determine and Estimate the MR Effects

Generally, two primary methods are used to determine the magneto-mechanical characteristics of MR materials: theoretical models and experimental methods. Experimental testing offers a more direct approach to characterizing the magneto-induced storage and loss moduli compared to theoretical models. Several studies have involved modifying conventional testing instruments to measure the magneto-induced moduli of MR materials. For example, Wang et al. [84] characterized the magneto-mechanical properties of MREs under compression by applying an axial magnetic field and mechanical loading. They designed a set of cylindrical coils with a lamellar chuck at the end of the coil core to facilitate clamping within the mechanical testing machine. The coil was made of copper wire and equipped with a highly permeable pure iron core of cylindrical section. These customized electromagnetic coils were clamped in the upper section of the universal testing machine to provide a magnetic flux density during the compression of the MR elastomers. The electromagnetic coil was used to study the effect of compression deformation on the magneto-mechanical properties, revealing the nonlinear magneto-mechanical characteristics of anisotropic MREs.

Zhao et al. [85] determined the dynamic mechanical behavior of MRE samples using a rotational rheometer equipped with an electromagnet. The tests were carried out in shear oscillation mode, and the electric current supplied to the electromagnet was varied in the range of 0–5 A. Finally, the relative MR effect of the samples was calculated using the fractional difference between the maximum field-on and the field-off moduli, divided by the field-off modulus. Ahmad Khairi et al. [86] also equipped a rotary rheometer with a current controller to generate the required magnetic field through the system, particularly around the test area. Dargahi et al. [87] designed a double-lap test setup to characterize the stress–strain behavior of MREs under static and dynamic shear loading. The shear fixture, made of a rigid polymer, held rectangular MRE specimens sandwiched between its inner and outer components. This assembly was secured to the testing system using two non-magnetic shafts: the upper shaft, fixed to the actuator, connected to the inner fixture member, while the lower shaft, attached to the outer member, was secured to a load cell on the fixed base. Permanent magnets, symmetrically positioned along the central axis, generated the required magnetic flux density, applying the field perpendicular to the specimen’s thickness. The fixture holding the magnets featured an adjustable spacing mechanism, allowing controlled variations in magnetic flux density. Additionally, the use of non-magnetic materials for the fixture and shafts minimized magnetic field leakage, ensuring accurate and reliable measurements.

Numerous theoretical models have also been developed to describe the magneto-induced moduli of MR materials. These models generally fall into two main categories. The first includes microscopic models, often based on magnetic dipole theory—reviewed in the next section—which focus on particle-level interactions [88]. The second includes macroscopic models, which comprise the continuum-based approach, the representative volume element (RVE) approach, and the phenomenological approach. These macroscopic models aim to capture the overall material behavior without explicitly resolving individual particle dynamics [89].

In the continuum-based approach used to predict the nonlinear magneto-mechanical behavior of MR solids, the effect of magnetic particles is assumed to be smeared within the elastomeric matrix. This results in a single equivalent medium that responds uniformly to external magnetic stimuli. These models describe the magneto-mechanical behavior of MR solids through coupled governing equations of elasticity (representing the matrix) and magnetism (representing the magnetic particles). The resulting magneto-mechanical governing equations are formulated to capture the response of MR solids under complex mechanical and magnetic loading and boundary conditions.

Although, to the best of the authors’ knowledge, the continuum-based models have not yet been applied to MR foams, various macroscale continuum-based models have been developed for MREs. Dorfmann et al. [90,91] were among the first to establish a continuum theory for MREs in which the magnetic field, rather than magnetization, is treated as the independent variable. They proposed a strain energy function that leads to a relatively simple constitutive equation. While their initial formulation was applied to isotropic MR solids, its general nature made it suitable to be used as a foundation for developing anisotropic theories. Nedjar [92] extended continuum-based theory to account for anisotropic mechanical responses by separating the contributions of the elastomeric matrix and the particle chains. Similarly, Kankanala et al. [93] developed a magneto-mechanical variational formulation for finite-strain magnetoelastic problems. Their approach derives both mechanical and magnetic field equations, as well as boundary conditions, by minimizing a total potential energy function. More recently, Akbari et al. [94] proposed a continuum model for the quasi-static magneto-mechanical behavior of MREs, in which the influence of magnetization on stiffness is incorporated into the shear modulus. Their model captures the coupled effects of magnetization, deformation, and particle-chain orientation within a single parameter, which effectively reduces the number of required invariants and material parameters, thus simplifying the constitutive formulation.

However, one limitation of continuum-based approaches is their inability to capture microstructural effects as MR solids are treated as homogeneous composites. Therefore, incorporating microstructural models alongside continuum-based models is recommended to obtain a more comprehensive understanding of MR material behavior.

Similar to the continuum-based approach, there is a lack of research on using RVE models to simulate the behavior of MR foams; however, several studies have applied this approach to MREs. Sun et al. [95] performed a finite element analysis of isotropic MREs under an external magnetic field and shear deformation using a 2D RVE configuration. However, they approximated the nonlinear magnetic flux density and magnetizing force of the magnetic particles using a linear model and assumed a relative permeability of 100 for CIPs. Xu et al. [96] conducted 3D modeling of isotropic MREs in tension under the magnetic field application. They used a linear material model to approximate the nonlinear hyperelastic behavior of the elastomeric matrix and a linear magnetic model for the CIP, thereby neglecting magnetic saturation. More recently, Abdollahi et al. [97] utilized both 2D and 3D RVE approaches to model MREs under the combined influence of an external magnetic field and pure shear deformation while accounting for both material and magnetic nonlinearities. They described the nonlinearity of magnetic particles using a magnetic flux density–magnetizing force curve that considers saturation. Their work also investigated the effects of factors such as CIP volume fraction, magnetic field intensity, and the mechanical behavior of the rubber matrix on the overall shear response. The results demonstrated the strong predictive capability of the developed 3D model in capturing the shear behavior of MREs.

Phenomenological models typically employ combinations of spring and viscous damper elements to characterize the magneto-induced shear moduli of MR materials. Examples include the Ramberg–Osgood model [98], and the modified Kelvin–Voigt viscoelastic model [99]. However, these models often have limited applicability, being effective only within specific frequency and magnetic field ranges. Furthermore, factors such as additive type and loading conditions introduce nonlinearities that make it difficult for theoretical models to accurately predict the magneto-induced shear storage modulus of MR materials across a wider range of conditions.

Although research on modeling the behavior of MR foams using microscopic and macroscopic models is still limited, recent studies have increasingly applied AI techniques, particularly machine learning models, to provide predictive capabilities for the behavior of MR foams. These AI-based approaches are discussed in Section 6. Before that, the following sections present a review of magnetic dipole theory and viscoelastic models, providing a deeper understanding of the theoretical frameworks traditionally used to study the MR effect in MR materials.

### 5.1. Magnetic Dipole Theory

The magnetic dipole model was among the first microscopic theoretical approaches used to explain the distribution of microscopic magnetic particles. Subsequently, more refined models have been developed, including the multi-chain model [100], the regular rectangular lattice model [101], and dipole-based models incorporating various chain structure angles [102]. While these models improve upon the basic dipole approach by accounting for more realistic particle arrangements, many remain idealized and are typically based on uniform, single-chain assumptions. This simplification limits their ability to be generalized to MR materials containing complex additives, such as surfactants, coatings, or secondary fillers, which alter interparticle interactions, introduce anisotropy, and disrupt regular chain formation. In such cases, the classical dipole model fails to capture the microstructural evolution and nonlinear behavior accurately. Multi-chain and lattice models offer improved predictive capability by considering inter-chain forces and spatial particle arrangements, which better reflect the modified microstructure and enhanced field response induced by additives.

Jolly and Carlson [103] presented a quasi-static, dipole-based model to describe the magneto-viscoelastic behavior of elastomer composites containing CIPs embedded within a polymer matrix. This model aims to analyze the magnetic field dependence of the composite’s elastic behavior. It assumes that the composite contains chains of magnetically permeable, spherical particles aligned by a strong magnetic field. The shear stress within these chains is attributed to interparticle magnetic forces. The particles interact via induced magnetic dipoles, and these interparticle forces contribute to a field-dependent shear modulus.

The analysis begins with the interaction energy (*E*_12_) between two adjacent dipoles with magnetic moments *m*_1_ and *m*_2_:

(11) $$E_{12}=\frac{1}{4\pi \mu_{1}\mu_{0}}\left[\frac{m_{1}\cdot m_{2}-3\left(m_{1}\cdot e_{r}\right)\left(m_{2}\cdot e_{r}\right)}{{\left|r\right|}^{3}}\right]$$

where $\mu_{0}$ is the magnetic permeability of free space, $\mu_{1}$ is the relative permeability of the medium, *r* is the center-to-center distance between the particles, and $e_{r}$ is the unit vector along the line connecting the dipoles. Assuming identical strength and direction for dipole moments, this energy expression simplifies. The stress induced by the application of a magnetic field can then be derived by taking the derivative of the interparticle energy density with respect to shear strain (ϵ):

(12) $$\sigma =\frac{\phi \epsilon \left(4-\epsilon^{2}\right)J_{p}^{2}}{8\mu_{1}\mu_{0}h^{3}{\left(1+\epsilon^{2}\right)}^{7/2}}$$

where ϕ is the particle volume fraction, $J_{p}$ is the induced particle polarization, and h is the mean particle spacing relative to the particle diameter. This model predicts that stress and modulus are highly sensitive to particle alignment, saturation polarization, and interparticle spacing. For small strains (ϵ < 0.1), we can rewrite the above equation as:

(13) $$\sigma =\frac{\phi \epsilon J_{p}^{2}}{2\mu_{1}\mu_{0}h^{3}}$$

For small strains (ϵ < 0.1), the composite modulus can thus be approximated as:

(14) $$G≅\;\frac{\phi J_{p}^{2}}{2\mu_{1}\mu_{0}h^{3}},\;\epsilon <0.1$$

The maximum field-induced modulus change occurs when the particles reach magnetic saturation and are proportional to ϕ. The model also incorporates a semi-empirical parameter to account for multipolar magnetic interactions and deviations from ideal chain structures.

The relationship between the average particle polarization ($J_{p}$) and the average flux density over the total area of composite (*B*) is derived by considering gradual saturation effects within the particle chains. Integrating the flux distribution yields the following expression for particle polarization:

(15) $$J_{p}=\frac{\frac{3}{2}\alpha^{3}B+\left(1-\alpha^{3}\right)J_{s}}{1+\frac{3}{2}\phi \alpha^{3}}$$

where α is a dimensionless parameter related to the particle saturation state and $J_{s}$ is saturation polarization of the particle material. Khanouki et al. [104] proposed that the average particle polarization of an MRE sample should be derived using the following updated equation:

(16) $$J_{p}=\frac{\frac{3}{2}\left(\alpha -\alpha^{3}\right)B+\left(1-\alpha^{3}\right)J_{s}}{1+\frac{3}{2}\phi \left(\alpha -\alpha^{3}\right)}$$

Nguyen et al. [99] utilized the interaction energy between two adjacent particles to calculate the force per unit volume in the x-direction $\left(F_{mx}\right)$ and y-direction $\left(F_{my}\right)$, corresponding to the horizontal and longitudinal force components, respectively. The expressions are given as follows:

(17) $$F_{mx}=\frac{J^{2}}{4\pi^{2}\mu_{1}\mu_{0}}\frac{3\left(5\sin^{2}\theta +4\right)\sin\theta }{r^{4}}$$

(18) $$F_{my}=\frac{J^{2}}{4\pi^{2}\mu_{1}\mu_{0}}\frac{3\left(5\cos^{2}\theta -3\right)\cos\theta }{r^{4}}$$

where $\left(\sin\theta =\frac{\varepsilon }{l}\right)$ and $\left(\cos\theta =\frac{r}{l}\right)$. Here, *l* represents the distance between the two magnetic dipoles, while ε and *r* denote the separation lengths between dipoles in the horizontal and vertical directions, respectively.

### 5.2. Viscoelastic Models

Viscoelastic models utilize combinations of ideal linear springs and Newtonian dashpots connected in parallel or in series (Kelvin–Voigt and Maxwell models) to describe the magnetoelastic component of MR solids. The linear spring represents the material’s elastic component, obeying Hooke’s Law by storing energy during deformation and releasing it upon stress removal for instantaneous recovery. Conversely, the Newtonian dashpot represents the viscous component, describing the material’s resistance to flow where stress is proportional to the strain rate. This element dissipates energy as heat and exhibits a time-dependent response. While these models typically involve a small number of parameters identifiable from experimental data, they often struggle to accurately represent frequency-dependent nonlinear properties. Increasing the number of components can enhance the model’s complexity, making parameter evaluation more challenging. This section will briefly describe some of these models.

#### 5.2.1. Kelvin–Voigt Model

This model represents viscoelastic behavior as a parallel arrangement of a spring and a dashpot. In this configuration, both the spring and the dashpot experience the same extension, while the total force is the sum of the forces in each element. Consequently, according to this model, the dashpot’s extension will always be equal to the extension produced in the spring. The constitutive equation for the Kelvin–Voigt [105] model under uniaxial stress is given by:

(19) $$\sigma \left(t\right)=E\epsilon \left(t\right)+\eta \frac{d\epsilon \left(t\right)}{dt}$$

where *σ*(*t*) is the stress as a function of time, *ϵ*(*t*) is the strain as a function of time, *E* is the elastic modulus, *η* is the viscosity coefficient, and *dϵ*(*t*)/*dt* is the strain rate. For shear deformation, the equation becomes:

(20) $$\tau \left(t\right)=G\gamma \left(t\right)+\mu \frac{d\gamma \left(t\right)}{dt}$$

where *τ*(*t*) is the shear stress, *γ*(*t*) is the shear strain, *G* is the shear modulus, *μ* is the shear viscosity, and *dγ*(*t*)/*dt* is the shear strain rate.

While the Kelvin–Voigt model provides a simple and often reasonably accurate representation of the viscoelastic behavior of materials, it has limitations when applied to MR foams. Firstly, the model assumes a linear relationship between stress and strain rate, which may not hold true for MR materials under large deformations or high strain rates [106]. Secondly, it cannot predict stress relaxation under constant strain, a phenomenon commonly observed in many viscoelastic materials [107]. Furthermore, the model predicts a constant storage modulus, which may not accurately reflect the behavior of MR foams, as their stiffness can exhibit some frequency dependence [108].

#### 5.2.2. Maxwell Model

In contrast to the Kelvin–Voigt model, the Maxwell model represents the viscoelastic behavior of materials as a series arrangement of a purely elastic spring and a purely viscous Newtonian dashpot [107]. Due to this series arrangement, the spring and dashpot experience the same stress, and the total strain of the material is the sum of the strains in each element.

The constitutive equation for a Maxwell model under uniaxial stress is given by:

(21) $$\sigma \left(t\right)+\;\frac{\eta }{E}\frac{d\sigma \left(t\right)}{dt}=\eta \frac{d\epsilon \left(t\right)}{dt}$$

For shear deformation, the equation becomes:

(22) $$\tau \left(t\right)+\;\frac{\mu }{G}\frac{d\tau \left(t\right)}{dt}=\mu \frac{d\gamma \left(t\right)}{dt}$$

While the Maxwell model offers advantages in describing stress relaxation, it also has limitations when applied to MR materials. Similar to the Kelvin–Voigt model, the Maxwell model assumes a linear relationship between stress and strain rate, which may not be valid for large deformations or high strain rates encountered in some applications [107]. Furthermore, the prediction of continuous viscous flow under constant stress does not accurately represent the behavior of MR solids, which typically exhibit a power-law pattern [109]. Although the model does predict a frequency-dependent storage modulus, the specific form of this dependence might not perfectly match the experimental behavior of MR solids across a wide frequency range [107].

#### 5.2.3. Zener Model (Standard Linear Solid Model)

This model is a three-parameter linear viscoelastic model that offers a more sophisticated representation of the viscoelastic behavior of materials compared to the simpler Kelvin–Voigt and Maxwell models. It combines elements of both Kelvin–Voigt and Maxwell models to capture phenomena such as creep with a finite limit and stress relaxation to a non-zero stress. The Zener model can be represented by a Maxwell element (a spring (*E*_2_, *G*_2_) and a dashpot (*η*_1_, *μ*_1_) in series) connected in parallel with another purely elastic spring (*E*_1_, *G*_1_). In this parallel arrangement, both the Maxwell element and the parallel spring experience the same total stress, and their strains are additive.

The constitutive equation for the Zener model under uniaxial stress is given by [110]:

(23) $$\sigma \left(t\right)+\;\frac{\eta_{1}}{E_{1}+E_{2}}\frac{d\sigma \left(t\right)}{dt}=\frac{E_{1}E_{2}}{E_{1}+E_{2}}\epsilon \left(t\right)+\frac{\eta_{1}E_{1}}{E_{1}+E_{2}}\frac{d\epsilon \left(t\right)}{dt}$$

Unlike the Maxwell model (which predicts unbounded creep) and the Kelvin–Voigt model (which does not predict stress relaxation), the Zener model provides a more realistic representation of the time-dependent behavior of MR solids [111]. Similar to the Kelvin–Voigt and Maxwell models, the Zener model is a linear viscoelastic model and may not accurately describe the nonlinear behavior observed in MR solids under large deformations or high strain rates. For a more accurate and comprehensive description of the viscoelastic behavior of MR solids, especially when dealing with nonlinearities or complex relaxation spectra, more advanced models such as the fractional derivative models may be necessary [109].

Chen et al. [111] developed a composite model to simulate the dynamic behavior of MREs. In their proposed model, the viscoelasticity of the polymer composite, magnetic field-induced properties, and interfacial slippage between the matrix and particles were modeled by analogy with a Zener (standard linear solid) model, a stiffness-variable spring, and a spring-Coulomb friction slider, respectively. They investigated how various factors influence MRE performance by changing parameters within the model. By substituting cyclic loading conditions into the constitutive relationships, they obtained hysteresis loops (shear stress vs. shear strain plots) and calculated dynamic properties such as shear modulus and loss factor. The simulation results were compared with expected MRE behavior and theoretical mechanisms. Specifically, simulations were performed for interfacial bond strengths ranging from 0.001 to 10 MPa at strain amplitudes of 0.01, 0.1, 0.5, and 1, while other parameters were held constant. They concluded that the proposed composite model can predict the dynamic behavior of MREs with varying volume fractions of iron particles and matrix material properties

Li et al. [89] presented a four-parameter linear viscoelastic model to describe the dynamic mechanical behavior of MREs under harmonic loading. This model aims to capture the field-dependent stiffness and damping properties of MREs in the pre-yield regime, where they exhibit linear viscoelastic behavior. The proposed four-parameter model extends the classical three-parameter standard solid model by adding spring element ($k_{b}$) in parallel. This additional spring represents the field-dependent modulus. The remaining components consist of two springs ($k_{1}$ and $k_{2}$) and a dashpot ($c_{2}$) arranged in series and parallel configurations to account for the material’s intrinsic viscoelasticity and damping. The stress–strain relationship, derived using linear viscoelasticity theory, results in a complex modulus ($G^{*}$):

(24) $$G^{*}=G_{1}+iG_{2}$$

where $G_{1}$ and $G_{2}$ are the real (storage modulus) and imaginary (loss modulus) components, respectively, and described as:

(25) $$G_{1}=\frac{\left(k_{1}k_{b}+k_{2}k_{b}+k_{1}k_{2}\right)\left[{\left(k_{1}+k_{2}\right)}^{2}+c_{2}^{2}\omega^{2}\right]+c_{2}^{2}\omega^{2}k_{1}^{2}}{\left(k_{1}+k_{2}\right)\left[{\left(k_{1}+k_{2}\right)}^{2}+c_{2}^{2}\omega^{2}\right]}$$

(26) $$G_{2}\;=\;\frac{c_{2}\omega k_{1}^{2}}{{\left(k_{1}+k_{2}\right)}^{2}+c_{2}^{2}\omega^{2}}$$

where $k_{b}$, $k_{1}$, $k_{2}$ and $c_{2}$ are model parameters, and ω is the excitation frequency. The model assumes harmonic strain inputs:

(27) $$\gamma \left(t\right)=\gamma_{0}\sin\left(\omega t\right)$$

with a corresponding stress response:

(28) $$\tau \left(t\right)=\gamma_{0}\sqrt{G_{1}^{2}+G_{2}^{2}}\sin\left(\omega t+\phi \right)$$

where $\phi =\tan^{-1}\left(G_{2}/G_{1}\right)$ represents the phase lag between stress and strain.

The model parameters ($k_{b}$, $k_{1}$, $k_{2},$ and $c_{2}$) are typically identified using a least-squares optimization method to minimize the error between the simulation and experimental data. Li et al. [89] used experimental data from MRE samples with 60 wt.% CIP content subjected to sinusoidal excitations at various strain amplitudes, frequencies, and magnetic flux densities (up to 440 mT). Their results showed that both the storage modulus (*G*_1_) and loss modulus (*G*_2_) increased with increasing magnetic field and excitation frequency, demonstrating the tunable stiffness and damping properties of MREs. The model predictions showed excellent agreement with the experimental results, validating the accuracy of the four-parameter model. The model’s ability to predict field-dependent viscoelastic properties makes it a valuable tool for designing MRE-based devices, such as tunable vibration absorbers and stiffness-controllable mounts. The study also highlights the influence of magnetic fields on the dynamic properties of MREs and provides insights into optimizing their performance under varying operational conditions.

Although widely used as foundational models in viscoelastic theory, classical viscoelastic models are built upon simplifying assumptions that limit their applicability to MR foams. They assume linearity, time-invariant material properties, and idealized, discrete mechanical elements (i.e., perfectly elastic springs and purely viscous dashpots). The Kelvin–Voigt model, for instance, assumes that stress is the sum of elastic and viscous components under simultaneous deformation, which restricts its ability to model stress relaxation behavior. Conversely, the Maxwell model captures stress relaxation but fails to adequately represent creep or bounded strain under constant stress [112]. These assumptions may hold for small deformations and low-frequency loading, but they become increasingly inaccurate for MR foams subjected to large strain amplitudes, high strain rates, and magnetic field-induced stiffening. Moreover, classical models treat the material response as a series or parallel combination of discrete elements, inherently lacking the memory-dependent behavior and continuous spectrum of relaxation times observed in MR foams. These foams exhibit nonlinear, field-dependent moduli, which are further influenced by porous microstructures, interfacial friction, and air–solid interactions—none of which are adequately captured by the idealized structure of classical models. Attempts to address these shortcomings using multi-branch generalizations of Maxwell or Voigt models lead to increased model complexity, over-parameterization, and reduced interpretability, especially when fitting experimental data over wide frequency ranges [112].

These limitations underline the need for more generalized and physically representative frameworks. In particular, fractional viscoelastic models, which use fractional derivatives to describe stress–strain relations, offer enhanced flexibility in modeling the complex, history-dependent, and tunable behavior of MR foams under magnetic fields. The following section explores these models in detail.

### 5.3. Fractional Derivative Viscoelastic Models

As mentioned earlier, the viscoelasticity of MR solids often exhibits nonlinear behavior as a function of frequency. The classical viscoelastic models, such as Kelvin–Voigt, Maxwell, and Zener models, which rely on ideal linear springs and viscous dashpots connected in parallel or series, often cannot accurately describe this nonlinear viscoelastic behavior of MR solids across a wide frequency range and strain rates [113]. While increasing the number of components in these classical models can improve their fit to experimental data, this also leads to greater model complexity and difficulties in parameter evaluation.

Fractional-order differential equations have emerged as a powerful tool for representing the frequency-dependent viscoelasticity of MR materials. This approach can model viscoelasticity with a single equation and a relatively simple algorithm. For instance, the fractional Maxwell model can capture more complex dynamic behavior than the standard, serially connected Maxwell model, with the fractional-order coefficient serving as an effective interpolation parameter [99]. However, it is important to note that the accuracy of fractional models is highly sensitive to the values of the model parameters. Although several methods have been proposed for determining these parameters, they often require extensive experimental data and significant computational effort to achieve optimal parameter values [114]. Therefore, further research is needed to develop simpler and more efficient procedures for parameter determination, which would enhance the practical applicability of fractional viscoelastic models. In this section, we will review several fractional derivative models that are suitable for describing the frequency-dependent viscoelastic behavior of MR solids.

#### 5.3.1. Fractional Kelvin–Voigt Model

To incorporate fractional-order derivatives into material constitutive equations, the Newtonian dashpots in classical rheological models can be replaced by a fractional derivative element, often referred to as a “spring-pot” element. The spring-pot element effectively combines solid-like (Hookean spring) and liquid-like (Newtonian dashpot) behavior through a fractional-order differential operator, as described below [55]:

(29) $$\sigma =G\;\tau^{\alpha }D_{t}^{\alpha }\gamma$$

In this expression, *σ*, *γ,* and *G* represent the shear stress, strain, and modulus, respectively. The parameter *τ* = *η*/*G* denotes the relaxation time, which is associated with the time required for segments of polymer chains to undergo reorganization and reorientation to reach a new structural equilibrium state. *η* is the material viscosity, and $D_{t}^{\alpha }\gamma$ is the fractional derivative of order *α* (with 0 ≤ *α* ≤ 10) of the strain with respect to time. When *α* = 0, the equation reduces to classical elastic behavior: $\sigma =G\;\tau^{0}D_{t}^{0}\gamma =G\gamma$. When *α* = 1, the model corresponds to Newtonian viscous flow: $\sigma =G\;\tau^{1}D_{t}^{1}\gamma =\tau D_{t}^{1}\gamma$.

Following the same concept, the constitutive equation for the fractional derivative Kelvin–Voigt model [105] is obtained by replacing the first-order time derivative in the classical Kelvin–Voigt model with a fractional-order derivative of order *α*:

(30) $$\sigma \left(t\right)=E\;\epsilon \left(t\right)+\eta D_{t}^{\alpha }\epsilon \left(t\right)$$

Values of *α* between 0 and 1 allow the model to represent viscoelastic behavior that lies between purely elastic and purely viscous responses. This capability is particularly useful for describing the behavior of materials such as MREs and MR foams, which exhibit both viscoelastic damping and energy dissipation, especially under the influence of a magnetic field. In the context of stress relaxation in MR solids, when a constant strain is applied, the classical Kelvin–Voigt model predicts an exponential decay of stress toward Eϵ. In contrast, the fractional derivative Kelvin–Voigt model can capture a power-law decay of stress over time—a behavior that has been observed in certain MREs and foams [115]. Although the fractional Kelvin–Voigt model provides enhanced accuracy in modeling viscoelasticity, it may still fall short in fully describing the complex behavior of MR materials under large deformations or complex loading conditions.

#### 5.3.2. Fractional Maxwell Model

The fractional Maxwell model extends the classical Maxwell model by incorporating fractional calculus, allowing a more accurate description of memory effects, stress relaxation, and time-dependent behavior, all of which are highly relevant to MR solids under magnetic fields. Similar to the fractional Kelvin–Voigt model, the first-order time derivative in the fractional Maxwell model is replaced with a fractional derivative of order *α* (0 ≤ *α* ≤ 1). The resulting model captures anomalous relaxation and non-local time effects.

The constitutive equation for the fractional Maxwell model [116] is:

(31) $$D_{t}^{\alpha }\epsilon \left(t\right)\;=\frac{1}{E}\;D_{t}^{\alpha }\sigma \left(t\right)+\frac{1}{\eta }\sigma \left(t\right)$$

where, by considering *α* = 1, the model reduces to the classical Maxwell model. MR solids exhibit stress relaxation, and the fractional Maxwell model can capture the details of this relaxation more accurately than the classical model. Furthermore, the fractional Maxwell model naturally incorporates frequency dependence in its dynamic response, which is crucial for MREs used in vibration control, where their behavior varies with the frequency of vibrations. Furthermore, fractional derivatives account for the “memory” of the material, meaning its current response depends on its past deformation history [99].

Nguyen et al. [99] developed a composite nonlinear model to describe the dynamic behavior of MREs in shear mode, consisting of three components: a fractional Maxwell viscoelasticity model to represent the frequency-dependent viscoelastic behavior with only three parameters; a magnetic dipole interaction model; and an adaptive smooth Coulomb friction model. This model effectively captured the nonlinear response of MREs over a broad frequency range (1–40 Hz) and shear strain amplitudes (5–20%). The simulation results showed good agreement with experimental data, achieving fitness values greater than 92% in most cases, thereby validating the model’s capability to accurately describe the complex dynamic characteristics of MREs.

#### 5.3.3. Fractional Zener Model

The classical Zener model can be modified by replacing the dashpot with two spring-pots in series, labeled a and b. The spring-pot “a” characterizes short-term viscoelastic behavior associated with high-frequency regions, while the spring-pot “b” characterizes long-term viscoelastic behavior in low-frequency regions. The two spring elements represent the elastic polymer behavior [55].

The differential equation of non-integer order for the fractional Zener model [117] can be written as:

(32) $${\sigma \left(t\right)+a_{1}D}_{t}^{\alpha_{1}}\sigma \left(t\right)\;=b_{0}\;\epsilon \left(t\right)+b_{1}D_{t}^{\beta_{1}}\epsilon \left(t\right)$$

where *α*_1_, *β*_1_ are the orders of the fractional derivatives, with 0 < *α*_1_ < 1 and 0 < *β*_1_ < 1.

Cordova et al. [55] fabricated isotropic and anisotropic MREs reinforced with 20% and 30% by weight of CIPs for quantitative analysis. They modeled the rheological response behavior of the experimental data using the fractional Zener model. Theoretical isothermal diagrams of *G*′ (*f*) and *G*″ (*f*) were found to describe the experimental data well when the fractional parameters *α* and *β* were appropriately adjusted. The Cole–Cole diagrams were plotted to determine the values of *α* and *β*, which served as an indirect measure of molecular mobility for mechanical relaxation phenomena by fitting theoretical predictions to experimental data. It was concluded that the fractional Zener model accurately predicts the isothermal spectra of the complex modulus for both isotropic and anisotropic MREs reinforced with CIPs.

Nadzharyan et al. [118] presented a detailed investigation into the dynamic mechanical behavior of MREs under varying magnetic fields using fractional rheological models. Recognizing the limitations of classical viscoelastic models in capturing the complex, field-dependent behavior of MR solids, especially at high particle loadings, they proposed a series of fractional-order models to account for the fractal-like microstructure and field-induced reconfigurations of magnetic fillers. Their study focused on MREs composed of silicone rubber matrices filled with 70–80 wt.% CIPs. Rheological tests were conducted in the linear viscoelastic regime using dynamic torsion oscillations under magnetic fields ranging from 0 to 800 mT. The authors identified three distinct regimes of magneto-mechanical behavior: First, at low magnetic fields, the material response is dominated by the elasticity of the polymer network. Second, in the intermediate field range, rapid reorganization of the filler network occurs, accompanied by significant increases in both the storage modulus (G′) and the loss modulus (G″). Third, at high magnetic fields, magnetic aggregates reach saturation, and the mechanical properties plateau.

**Figure 3:**
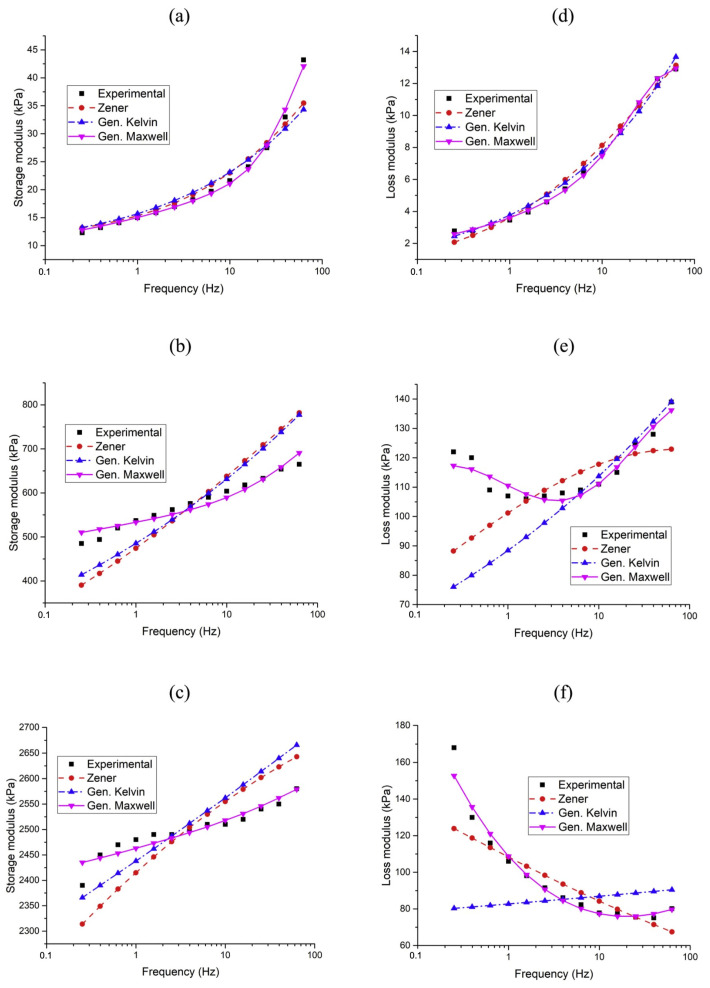
Experimental data and fitting curves for the frequency dependences of the storage modulus (**a**–**c**) and the loss modulus (**d**–**f**) of an MRE sample containing 75 wt.% CIP for three magnetic field values: 40 mT (**a**,**d**), 200 mT (**b**,**e**), and 800 mT (**c**,**f**). This figure is reproduced from reference [118] with permission from John Wiley and Sons.

To model these behaviors, they tested six fractional viscoelastic circuits, including extensions of classical Maxwell, Kelvin–Voigt, and Zener models. Notably, they found that simple one-fractional-element models could adequately capture behavior at either low or high field strengths but failed to represent the intermediate regime. In contrast, two-fractional-element models, particularly the fractional generalized Maxwell model, provided excellent agreement across the entire range of magnetic fields. These models were capable of separately characterizing the contributions of the polymer matrix and the magnetic filler network, including their interactions and restructuring dynamics. The fractional-order parameters (α, β) were extracted through a model fitting procedure that minimized an error function representing the difference between experimental and theoretical frequency dependences of the dynamic modulus. This minimization was performed using numerical optimization algorithms, specifically the conjugate gradient method and the quasi-Newton method, while adhering to physical constraints on the parameters (e.g., fractional orders between 0 and 1, and positive viscoelasticity coefficients and elastic moduli). These parameters varied systematically with the applied magnetic field. These changes were interpreted as indicators of microstructural evolution, offering a phenomenological yet physically meaningful link between macroscopic rheology and the internal morphology of MREs. Figure 3 illustrates their experimental data and model fitting curves for three different models, including the Zener model, generalized Kelvin–Voigt model, and generalized Maxwell model, depicting the frequency dependence of the storage modulus and loss modulus of an MRE sample containing 75 wt.% CIP under magnetic fields of 40 mT, 200 mT, and 800 mT.

While fractional viscoelastic models offer significant advantages in modeling the broadband, field-tunable behavior of MR foams, their practical implementation poses several challenges. First, the physical interpretation of fractional parameters (e.g., fractional orders α and β) is often less intuitive than classical mechanical constants, making model validation and comparison difficult [118]. In the context of MR foams, this is further complicated by the heterogeneous structure and multiphase interactions between the polymer matrix, magnetic particles, and gas-filled pores. Moreover, the accuracy of fractional models heavily depends on the availability of high-resolution experimental data across a wide frequency and strain range [114]. This is essential to capturing the full spectrum of relaxation dynamics and to robustly fit fractional parameters using inverse optimization techniques. Field-dependent behavior of MR foams introduces nonlinearity and history effects, requiring multiple datasets under varying magnetic field strengths, amplitudes, and loading frequencies. Without this extensive data, parameter identification may be ill-posed or result in overfitting with limited predictive power [114].

### 5.4. Einstein–Guth–Smallwood Model

The complex modulus of elastomers filled with randomly dispersed, rigid, spherical particles (either non-magnetic or magnetic but in the absence of an applied magnetic field) at a volume concentration *ϕ* can be estimated using the Einstein–Guth–Smallwood model [34]:

(33) $$E^{*}=E_{0}\left(1+2.5\phi +14.1\phi^{2}\right)$$

where *E*_0_ is the complex modulus of the unfilled elastomer. This equation is applicable to estimating the complex modulus of MREs when no magnetic field is applied. Wereley et al. [34] adapted the Einstein–Guth–Smallwood model to study the strain-dependent dynamic compressive properties of MR foams, developing an experimental framework for characterizing their behavior. They extended the model to incorporate the influence of both particle concentration (*ϕ*) and magnetic field strength (*B*) by introducing an additional term. The complex modulus under an applied magnetic field *B* is then given by:

(34) $$E^{*}=E^{0}\left[1+2.5\phi +14.1\phi^{2}+\alpha {\left(\phi B\right)}^{\beta }\right]$$

where *α* and *β* are empirical constants determined through curve fitting of experimental data.

The Einstein–Guth–Smallwood model remains a foundational approach to estimating the effective modulus of MR materials by linking microstructural features—such as particle volume fraction—to macroscopic stiffness. However, its applicability to MR foams is limited by assumptions of homogeneous dispersion, idealized matrix behavior, and linear magnetic responses, which do not fully capture the complex field-dependent deformation and porosity-driven heterogeneity present in foam structures. In this context, AI-based methods can serve as a complementary tool for analyzing and predicting the behavior of MR foams under varying experimental conditions [30]. For instance, machine learning algorithms can be employed to identify dominant features influencing the modulus from large experimental datasets, validate theoretical trends predicted by models like Einstein–Guth–Smallwood, or explore regimes where deviations from model assumptions occur. This can guide model refinement or suggest appropriate ranges of applicability. Additionally, AI can support the inverse identification of microstructural parameters (e.g., effective volume fraction) based on experimental input–output data, thus helping calibrate theoretical models more efficiently [119]. These tools are especially useful when working with large datasets or when exploring parameter regimes where classical assumptions may break down. Further details on the application of AI approaches to modeling MR foam behavior are presented in the following section.

### 5.5. Physics-Based Computational Methods in the Modeling of MR Materials

Physics-based computational techniques such as density functional theory (DFT), molecular dynamics (MD), and Monte Carlo (MC) simulations have long served as foundational tools in materials science, offering detailed insights into material behavior across multiple scales. Although their direct application to MR foams remains relatively limited compared to bulk polymers or MREs, these methods hold significant potential for elucidating structure–property relationships in MR foams from first principles.

Density functional theory [120], a quantum mechanical method, is primarily used to investigate the electronic structure of atoms and molecules. In the context of MR foams, DFT is particularly valuable for understanding the intrinsic properties of magnetic filler particles, which determine their magnetic response and interfacial interactions with the polymer matrix. Additionally, DFT has been used to explore surface functionalization strategies aimed at improving particle dispersion or enhancing particle–matrix bonding, both of which are critical for tailoring the magneto-mechanical properties of MR foams.

Molecular dynamics simulations offer a complementary mesoscale perspective by modeling the time-dependent behavior of atoms and molecules based on Newtonian mechanics. This approach is well suited for studying interfacial mechanics between filler particles and polymer chains, especially in MR foams where dispersion, agglomeration, and matrix stiffness significantly influence performance. MD has also been employed to investigate the formation of particle networks during magnetic curing, the mechanical response of polymer cell walls under dynamic loading, and local viscoelastic effects at the microscale. For example, Lazim et al. [121] used MD to study stress relaxation behavior in MREs under constant strain, while Pei et al. [122] applied it to explore squeeze-strengthening effects on the rheological and microstructural behavior of MR fluids. However, despite these advances, limitations in the accessible time and length scales currently restrict MD’s ability to simulate the full complexity of foam architectures.

Monte Carlo methods, based on probabilistic sampling, are particularly effective for evaluating equilibrium properties, spatial distributions, and phase transitions. In the context of MR foams, MC simulations can be used to model random or field-aligned particle arrangements, investigate the statistical distribution of magnetic fillers in porous matrices, and estimate effective magnetic permeability under different loading conditions. Although MC techniques do not capture dynamics directly, they are efficient in exploring configuration spaces that are otherwise computationally intractable. Yongzhi et al. [123], for instance, used MC simulations in MR fluids to demonstrate particle relocation and structure formation under magnetic fields. Their results showed the emergence of chain-like and columnar particle structures aligned with the direction of the applied magnetic field, consistent with experimental observations. However, the application of such approaches to MR foams remains largely unexplored, representing a promising opportunity for future research.

While physics-based simulations such as DFT, MD, and MC offer rigorous, mechanistically grounded insights into MR foam behavior at various scales, they are often constrained by high computational costs, limited scalability, and the complexity of capturing the multi-scale and multi-physics nature of porous smart materials. Consequently, modeling entire MR foam systems using these approaches remains challenging. Nevertheless, these methods are indispensable for parameterizing continuum models, validating theoretical predictions, and generating synthetic data to inform machine learning models. To overcome the methods’ individual limitations, AI and machine learning techniques have emerged as powerful complements or alternatives. These data-driven approaches enable rapid prediction of material properties, uncover hidden structure–property relationships, and support the inverse design of MR foams, particularly when experimental data are sparse or traditional simulations are computationally prohibitive. Recent research increasingly focuses on integrating physics-based simulations with AI to leverage the strengths of both domains. The following section explores this growing role of AI in the modeling, optimization, and discovery of MR materials.

## 6. Artificial Intelligence in the Development and Optimization of MR Materials

While experimental methods have significantly advanced the development of MR materials, they are often time-consuming and labor-intensive. Furthermore, theoretical models, such as viscoelastic and magnetic dipole models, although valuable, struggle to accurately capture the complex, nonlinear behavior of these materials, especially under varying magnetic fields, excitation frequency, and strain conditions. These models frequently rely on simplifying assumptions and empirical fitting, which can limit their accuracy in real-world applications. Therefore, a more efficient and accurate method for predicting the magneto-induced mechanical properties of MR materials is essential.

Artificial intelligence has emerged as a transformative tool in the development and understanding of MR materials. Leveraging machine learning algorithms, predictive modeling, optimization techniques, and big data analytics, AI enables researchers to address challenges in material design, development, characterization, and application of MR materials. AI models can predict rheological properties, optimize material composition and microstructure, and improve manufacturing processes through data-driven approaches. Future advancements in AI—such as generative design, real-time monitoring, and enhanced simulations—offer capabilities that go beyond current modeling and optimization techniques. Generative design, for instance, uses AI to autonomously explore a vast solution space and propose novel material architectures optimized for specific performance targets, which is particularly useful in tailoring MR foam structures for damping or tunability. Real-time monitoring integrates sensor data with adaptive AI models to dynamically adjust system behavior, enabling closed-loop control of MR foam-based devices under variable operating conditions. Enhanced simulations driven by AI can significantly reduce computational cost while capturing complex, nonlinear responses across multi-scale material features. Collectively, these approaches accelerate discovery, improve cross-disciplinary integration (e.g., combining materials science with control systems), and enable the development of customized MR foam systems adapted to specific functional or environmental requirements. These advancements will ensure that AI remains at the forefront of innovation in the field of MR materials. This section reviews studies that have employed AI tools to investigate and develop MR materials.

### 6.1. Machine Learning and Deep Learning in MR Materials Study

Among the diverse tools within AI, machine learning, and especially deep learning, have recently become prominent in materials and mechanical engineering, particularly in the study of MR materials. A key application is the development of predictive models. These models are then often used to optimize parameters, aiming to maximize or minimize desired properties of MR materials. Previous applications of machine learning include predicting the viscosity and shear stress of MR fluids [119,124], modeling the tensile/compression behavior of MREs [125], and analyzing MR device behavior [126,127,128,129]. Machine learning models offer a data-driven approach, trained on large datasets to extrapolate future performance [87]. They offer advantages over parametric models by capturing nonlinear input–output relationships without explicit parameter identification, which is particularly beneficial for studying the inherent nonlinear behavior of MR materials. A key advantage of machine learning is that it requires no prior assumptions about the functional relationship between inputs and the target variable.

Sarath et al. [130] employed a hybrid approach that combined an artificial neural network (ANN) with a genetic algorithm (GA) to optimize the parameters of an MR fluid foam damper for maximum damping force. Their methodology involved collecting a dataset of damping force response data from 30 different MR fluid foam damper configurations, derived from experimental studies incorporating 5 PU foam densities, 3 iron particle sizes, and 2 oil grades. A multilayer normal feedforward network with a 3-4-1 configuration was utilized, trained using the Levenberg–Marquardt algorithm. This specific configuration, determined through rigorous experimentation and a trial-and-error approach for optimal performance, consisted of three input neurons (representing foam density, iron particle size, and oil grade), four neurons in the hidden layer, and one output neuron (for damping force). Its effectiveness was evidenced by a high regression coefficient (R = 0.99652), indicating a strong correlation between experimental and predicted outputs. The Levenberg–Marquardt training algorithm minimizes the cost function by using the Gauss–Newton method combined with the gradient descent method to update the weight and bias of the network. The output of the ANN model served as the fitness function for the GA, which generated a population of solutions and iteratively refined them through selection and genetic operators (crossover and mutation). The ANN model identified foam density as the most influential factor in determining the damping force (approximately 55.08%), followed by iron particle size (43.21%), with oil grade contributing minimally (1.71%). The optimized parameters obtained through GA and ANN led to an increase in the damping force. Specifically, the damping force increased from an average of 242.2 N (calculated from 30 sets of data) to a maximum optimized value of 299.7 N, representing a 57.5 N gain or an approximate 23.7% improvement.

Peng et al. [131] explored an AI-driven approach to optimize MR fluid preparation. They implemented an enhanced grey wolf optimization (GWO) algorithm to refine the hyperparameters of a least squares support vector machine (LSSVM). Initially, three critical process parameters—ball milling time, silicone oil viscosity, and nano-magnetic particle volume fraction—were identified as input variables for the predictive model. A dataset comprising 15 MR fluid samples, each prepared with varying combinations of these parameters, was generated. Subsequently, the improved GWO-optimized LSSVM was utilized to predict the influence of the process parameters on MR fluid performance, specifically shear yield stress, zero-field viscosity, and sedimentation rate. The findings revealed that the optimized LSSVM exhibited superior predictive accuracy for MR fluid properties. Although this approach incurred an increased computational time, typically ranging between 2 and 20 s, this is generally not considered a significant trade-off for many real-world applications (e.g., in damping systems, precision actuators, or real-time control applications) where enhanced predictive accuracy is critical.

Dhiman et al. [132] investigated the influence of CIP concentration and magnetic field strength during curing on the rheological properties of MREs, employing an ANN model whose hyperparameters—such as hidden layer size and training function—were optimized using Bayesian optimization to minimize the mean squared error (MSE) of predictions. A total of 16 MRE samples were prepared with varying CIP concentrations (5, 10, 15, and 20%) and cured under different magnetic field strengths (0, 0.15, 0.3, and 0.5 T). These samples were analyzed through amplitude, frequency, and magnetic sweep tests, with their storage (*G*′) and loss moduli (*G*″) measured to train and test the AI model. The ANN model consisted of 5 input neurons, a hidden layer with 1 to 100 neurons (with 41 neurons yielding the lowest MSE), and an output layer with 2 neurons representing the storage and loss moduli of the MRE samples. The study found that increasing CIP content enhanced the dynamic modulus of MREs at a fixed strain. However, as the strain increased from 0 to 20%, the storage modulus decreased by approximately 70% for MREs with a 20% CIP volume fraction, whereas it dropped by only 25% for those with 5% CIP. The optimized ANN model, trained with 70% of the dataset, accurately predicted the behavior of MREs under various conditions, achieving R^2^ values of 0.97 for storage and loss modulus on unseen data.

Rabbani et al. [124] investigated the rheological properties of MR fluids using ANN and support vector regression (SVR). They modeled the effects of temperature, magnetic field strength, and shear rate on shear stress using 54 selected experimental data points from a dataset of 600. The MLP network structure consisted of three input variables, a single hidden layer with seven neurons, and one output variable. The Levenberg–Marquardt optimization algorithm was used, yielding an optimal network with a correlation coefficient of 0.995 and a root-mean-square error (RMSE) of 0.035. In SVR modeling, the Gaussian kernel produced the best results, with model parameters determined through trial and error, achieving an RMSE of 0.0499 and an R^2^ value of 0.9991.

Comparing the models’ predictions with experimental data across various temperatures and magnetic field strengths showed that, at low shear rates, the SVR model more reliably predicted the flow curve, particularly for dynamic yield stress. In contrast, the ANN model struggled to accurately predict shear stress at low shear rates. Furthermore, both models indicated that at very low shear rates, the shear stress approaches a constant dynamic yield stress. While the Herschel–Bulkley model proved inadequate for predicting dynamic yield stress over a wide range of temperatures and magnetic field strengths, the SVR model provided reliable predictions in both interpolation and extrapolation, closely matching experimental data.

Zhao et al. [126] proposed a backpropagation ANN (BP-ANN) model to characterize the nonlinear hysteresis of MRE isolators for vibration control. Using displacement, velocity, and applied current as inputs, and transmitted force as the output, they trained and tested the BP-ANN model with experimental data. They demonstrated that a 3-8-1 configuration with a sigmoidal activation function accurately approximated the MRE isolator’s performance, exhibiting a small error range. They concluded that the BP-ANN model could be beneficial for online control of MRE isolators in semi-active vibration control systems.

Saharoddin et al. [133] developed a predictive model for estimating the storage modulus and loss modulus of MREs based on excitation frequency, magnetic flux density, magnetic particle weight percentage, and curing magnetic field, under both isotropic and anisotropic conditions. The model was trained using two machine learning approaches: extreme larning machine (ELM) and ANN. Specifically, the ANN employed a multilayer perceptron (MLP) architecture and was trained using the Levenberg–Marquardt (LM) algorithm, a variation of the BP learning method. The study utilized a dataset comprising 2160 data points across 70 datasets, with 80% allocated for training. Various network configurations were explored by varying activation functions and the number of hidden nodes. The performance of the BP-ANN and ELM models was evaluated against experimental results using RMSE and the coefficient of determination (R^2^). Among the tested configurations, the ANN model with eight hidden nodes and the ELM model with a sine activation function and 55,000 hidden nodes achieved the highest accuracy. The ANN model demonstrated greater consistency across both storage and loss modulus predictions. However, errors increased with higher magnetic field strength, and isotropic distributions exhibited larger prediction errors compared to anisotropic ones. While ELM outperformed ANN in terms of overall accuracy and training efficiency, ANN provided better generalization when predicting learned data.

Rohim et al. [30] developed ANN and ELM models to predict magnetostriction behavior (strain and normal force) in MR foams with varying CIP compositions. Both models used a single hidden layer feedforward neural network (SLFN) architecture. These models captured the nonlinear relationship between CIP composition, magnetic field, strain, and normal force. For the ANN, RMSProp and ADAM learning algorithms with sigmoid and ReLU activation functions were used. The ELM model employed hard limit, ReLU, and sigmoid activation functions. The models were evaluated on both training and testing data. The ANN with RMSProp and sigmoid activation, and the ELM with ReLU and hard limit activation functions, demonstrated higher accuracy than other models. Correlation analysis and comparison with experimental data showed that the ELM with hard limit activation was more generalized in predicting strain and normal force, achieving an R^2^ of 0.999 and an RMSE of less than 0.002. Based on RMSE and R^2^ values, the ANN (RMSProp, sigmoid), ELM (ReLU), and ELM (hard limit) models demonstrated higher accuracy in predicting magnetostriction behavior. The authors concluded that the ELM (hard limit) model could be used to predict the magnetostriction behavior of MR foams with different CIP compositions, which is valuable for material development and the design of MR foam devices, particularly in soft robotics applications.

Ren et al. [134] proposed a physics-guided deep learning model, magnetic dipole theory and CNN-BiLSTM-Attention (MD-CBA), for predicting the magneto-induced shear storage modulus of MREs. Twelve types of MRE samples were prepared, and their magneto-induced shear storage moduli were measured to train the MD-CBA model. First, the raw data were processed using the magnetic dipole model to obtain an initial estimate of the magneto-induced storage modulus. Second, the data underwent feature embedding, and local patterns were extracted using a CNN, representing the effects of material composition and magnetic field strength on the storage modulus. Third, a BiLSTM network was employed to capture the sequential relationship between the storage modulus and the applied magnetic field. Finally, the Attention mechanism assigned weights to the most influential factors affecting the storage modulus. The hyperparameters of the model were manually selected. The MD-CBA model accurately predicted the magneto-induced shear storage modulus of MREs, achieving an average R^2^ of 0.99. Even with the addition of silicone oil and varying loading frequencies, the model maintained high accuracy, achieving R^2^ values of 0.968 and 0.985, respectively.

### 6.2. Applications of AI in Material Discovery

While researchers typically use AI to predict material properties as a function of variables like composition, Bahiuddin et al. [119] presented an inverse model for MR fluids, predicting composition based on magnetic field-dependent rheological properties. They employed ELM and BP techniques to train feedforward neural network (FFNN) models. The model outputs include the particle weight percentage, milling time (representing particle shape), and particle size. Predictions are based on three different input topologies, which consider factors such as yield stress in the on-state, yield stress in the off-state, magnetic field strength, and the slope of yield stress as a function of the magnetic field. A total of 3 experimental datasets, comprising 67 data points, were collected from various literature sources to establish correlations between yield stress, magnetic field, and composition. The first dataset consists of MR suspensions with varying particle shapes produced through milling. The second dataset focuses on MR grease, where different weight percentages of CIPs are used. The third dataset involves MR fluids with varying particle sizes. Simulation results, correlation analysis, and variable impact assessments indicate that the slope of the yield stress (*m*) plays a crucial role in the model’s accuracy, as it encapsulates information about yield stress and material magnetic properties. Additionally, including the off-state yield stress as an input improves model performance if it has a high correlation with the output. The model achieved an R^2^ value exceeding 0.80 for both training and testing datasets, demonstrating its effectiveness in predicting MR fluid compositions based on rheological properties.

Leng et al. [135] developed an ANN approach to predicting and analyzing the highly nonlinear behavior of an MRE isolator operating in shear/compression mixed mode. They proposed an ANN optimized by a fuzzy algorithm (ANNOFA) to approximate the nonlinear functional relationship between the inputs (displacement, velocity, frequency, and current) and the output (force) using a three-layer network with eight hidden neurons. The fuzzy logic system employed in this study is based on the Takagi–Sugeno fuzzy model, which decomposes complex nonlinear systems into simpler linear models valid within specific fuzzy regions. For performance comparison, they evaluated the proposed ANNOFA model against other existing models, including a backpropagation neural network, a viscoelastic k-c model, and a hysteresis Bouc–Wen model. The results demonstrated that ANNOFA outperformed these models in accurately capturing the nonlinear and hysteresis behavior of the MRE isolator.

## 7. Effect of Different Parameters on MR Effect and MR Foam’s Structure

The MR effect and matrix structure are influenced by a variety of parameters, which determine the performance and adaptability of MR materials in different applications. These parameters can be broadly classified into two categories: material-related parameters and operational parameters. Material-related parameters include pore characteristics (size, shape, etc.), the composition and concentration of magnetic particles, particle properties (e.g., size distribution, density, and shape), matrix properties (e.g., viscosity, elasticity), the incorporation of additives and surface treatments. Operational parameters encompass the strength and uniformity of the applied magnetic field, temperature, shear stress rate, and frequency of operation, among others. Among these operational parameters, the strength of the magnetic field is the most influential, making the MR effect a highly controllable and tunable mechanism. This section reviews key studies that have investigated the influence of different parameters on the MR effect in MR foams, providing insights into their roles in optimizing MR material performance.

### 7.1. Pore Characteristics

A key characteristic that distinguishes MR foams from other MR materials is their porosity. Plachy et al. [136] investigated the impact of porosity on the magneto-mechanical behavior of MR elastomers by incorporating a commercial azodicarbonamide-based foaming agent at two different loading levels. They compared these porous MR elastomers to a non-porous counterpart. Notably, under an applied magnetic field intensity of 1000 kA/m, the storage modulus increased from 280 kPa to 291 kPa and 418 kPa by increasing the foaming agent content from 0 to 4 and 6 parts per hundred rubber (phr), respectively. However, the mean pore size and pore number were not reported in their study. During single-strain measurements with the increasing and decreasing magnetic field, the MR foam also showed lower mechanical hysteresis compared to the non-porous material. This is attributed to the pores, which increase the interparticle distance and thus weaken the magnetic network formed at high magnetic fields. Porous MR materials can be potentially useful in applications where conventional MREs are too stiff, bridging the property gap between MR fluids and solid elastomers.

Ju et al. [72] investigated the influence of porosity on the magnetorheological characteristics of MR foams by incorporating ammonium bicarbonate (NH_4_HCO_3_) as a foaming agent into a silicone rubber matrix. Porosity was controlled by varying the NH_4_HCO_3_ content, which decomposes into CO_2_ during curing, forming a porous structure. Their results revealed that as the NH_4_HCO_3_ content increased from 0 to 6 wt.%, porosity increased accordingly, leading to a steady decrease in storage modulus. Specifically, as the NH_4_HCO_3_ content increased from 1 to 2, 4, and 6 wt.%, the porosity increased accordingly from 9 to 13, 28, and 35% (obtained using microstructure image processing), and the number of pores increased from 263 to 350, 480, and 525 pores (obtained using microstructure image marking). This steady increase in porosity and the pore number directly correlated with a decrease in storage modulus. For instance, under a 300 mT magnetic field, the MR foam with 35% porosity (525 pores) exhibited a lower storage modulus (G′ = 0.46 MPa) compared to the samples with 28% porosity (480 pores) and 13% porosity (350 pores), which had storage moduli of *G*′ = 0.48 MPa and 0.68 MPa, respectively. However, under a 600 mT magnetic field, the porous sample showed a remarkable 170% increase in storage modulus, while the non-porous sample increased by only 25%, indicating higher field sensitivity in porous structures. The study also showed that the loss factor increased with higher porosity, while the stress amplitude decreased significantly. In the first set of samples with 70 wt.% CIP, the stress amplitude dropped from 121.6 to 38.1 kPa—a 68.7% reduction—as the NH_4_HCO_3_ content increased from 0 to 6 wt.%. This enhanced sensitivity to magnetic fields in porous samples was attributed to weaker particle–matrix compatibility and increased interfacial friction caused by pore formation.

**Figure 4:**
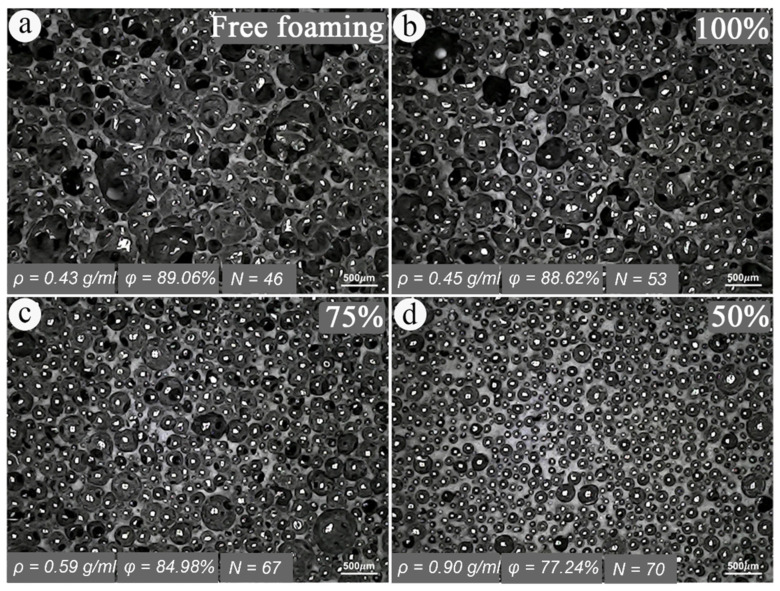
The micrographs of MR foam fabricated via (**a**) free foaming (sample A), (**b**) 100% constrained volume foaming (sample B), (**c**) 75% constrained volume foaming (sample C), and (**d**) 50% constrained volume foaming (sample D) at various mold lengths. For each sample, the corresponding density (*ρ*), porosity (*φ*), and estimated number of nucleated pores (*N*) are indicated within the respective sub-figures, demonstrating the effect of constrained foaming on the foam morphology. This figure is reproduced from reference [66] under Creative Commons CC-BY license.

Marzuki et al. [66] investigated enhancing the storage modulus of MR foams by introducing constrained foaming during fabrication. They reported that a 50% decrease in the length of the mold during foaming resulted in an increased density from 0.43 g/mL to 0.9 g/mL and an increase in the number of pores from 5 pores per unit area to 16 pores (Figure 4). While the precise numerical change in individual pore size was not explicitly reported, it can be inferred that the pores became smaller, as supported by the increase in density (*ρ*) and estimated number of nucleated pores (*N*), coupled with a decrease in overall porosity (*φ*), as illustrated in Figure 4. Reducing the mold volume by 50% increased the storage modulus by approximately 50% compared to free-foaming MR foam in the off-state condition. Under the application of an applied magnetic field, the absolute MR effect increased to 8.4 kPa with 50% constrained foaming, compared to 5.2 kPa with free foaming. They believed that these enhancements are attributed to the increased relative density and reduced MR foam porosity, which significantly promotes the formation of stronger chain-like structures.

In another study, Marzuki et al. [108] again investigated MR foams with 75 wt.% CIPs, prepared via an in situ polymerization method under two conditions: free foaming and constrained foaming with varying volumes. Morphological analysis of the foam pores, conducted using 3D laser microscopy, revealed a considerable reduction in the average pore diameter for the constrained samples. This was accompanied by improved homogeneity and uniformity. The average pore diameter for the sample without constrained foaming was 275 μm, which decreased to 228, 207, and 172 μm for samples with 100%, 75%, and 50% constrained foaming, respectively. The MR foams prepared under 50% constrained volume (which exhibited the most uniform pore size distribution) showed significant enhancements in the elastic moduli (*E*′ and *G*′) and the loss factor (*tan δ*). Under dynamic mechanical compression analysis, introducing constrained foaming (100% constrained volume) resulted in approximately a 25% increase in the initial *E*′ compared to the free-foaming sample. Further volume reductions (75% and 50% constrained foaming) led to increases of about 125% and 300%, respectively. A similar trend was observed in the rheological analysis, where a 13% increase in *G*′ was noted with the introduction of constrained foaming.

Some studies have reported that the morphological structure of MR foams can be regulated by manipulating the alignment of particles using time-dependent magnetic fields during the fabrication process [137,138]. However, the resulting MR foam would be classified as an anisotropic MR foam, as opposed to an isotropic type. Furthermore, manipulating the arrangement of CIPs within the foam matrix is another approach to enhancing the storage modulus of MR foam. However, further research is needed to investigate the specific effects of porosity characteristics, such as size and distribution, on the MR effects and overall structure of MR foams while keeping other aspects, like magnetic particle volume fraction, constant.

While significant progress has been made in characterizing the morphological properties of MR foams, particularly concerning the influence of fabrication methods like constrained foaming on density, porosity, and pore number, several critical gaps remain in the literature. A more profound understanding is needed regarding the detailed mechanistic explanation of how the specific distribution of pores (beyond general compactness) affects the overall mechanical and magnetorheological properties. Current studies often acknowledge this relationship but lack explicit analyses of the underlying physical mechanisms. Furthermore, there is a clear absence of explicit quantification of the links between enhanced particle–matrix compatibility or interfacial friction and specific performance metrics like stress amplitude. Such detailed correlations are crucial for optimizing material design. Complementing this, a rigorous and detailed statistical analysis on the distribution of pore sizes is also largely under-reported. Implementing such analyses would significantly strengthen the claims regarding the precise influence of porosity on the resultant mechanical and magnetorheological properties.

### 7.2. Effect of Magnetic Particles on MR Effect

The concentration of magnetic particles within the foam matrix is crucial for determining MR foam properties as these particles are mainly responsible for the MR effect. Norhaniza et al. [139] reported that increasing the CIP concentration in MR foams fabricated via an in situ method leads to an increase in magnetic saturation and remanence. This enhancement arises from a higher magnetic moment density and more frequent interparticle contact as the particle volume fraction increases. Such contact reduces grain boundaries and allows CIPs to behave as larger magnetic domains, thereby enhancing magnetic field induction and increasing net magnetization. As a result, the MR foam exhibits improved sensitivity to the magnetic field, along with increased saturation strain.

However, their study also observed that this effect tends to saturate at higher concentrations. Specifically, 75 wt.% CIPs yielded the highest magnetostrictive strain (1.66% at 0.45 T), but further improvement beyond this level is unlikely due to structural and physical limitations within the composite. As CIP concentration increases, several concurrent mechanisms begin to constrain further performance enhancement. First, excessive CIP loading reduces the polymer matrix content, lowering matrix elasticity and hindering the deformation capacity of the foam. Second, closely packed particles have limited ability to rotate or align into chain-like structures under a magnetic field, diminishing the formation of stress-inducing particle chains. Norhaniza et al. [139] further modeled this behavior using Langevin-type magnetization theory, where magnetization asymptotically approaches a maximum with increasing magnetic field and particle content. Additionally, the magnetically induced internal force is proportional to the square of the magnetic moment, while the resulting mechanical stress in the matrix depends on the interparticle distance. As this distance decreases, structural constraints begin to dominate. Thus, although magnetic saturation continues to rise, the mechanical compliance of the composite decreases, leading to a saturation of the MR effect.

Ju et al. [72] studied the effect of increasing magnetic particle concentration on the shear storage modulus of a foam composed of CIPs, silicone rubber, and ammonium bicarbonate (NH_4_HCO_3_). Their study revealed a pronounced and progressive increase in shear storage modulus with higher CIP loading, both in the absence and presence of an applied magnetic field. For instance, under zero magnetic flux density, the shear storage modulus was measured at 0.39 MPa, 0.49 MPa, and 0.78 MPa for 60, 70, and 80 wt.% CIP, respectively. They explained this increase by stating that MRE is a composite material, and its storage modulus can be estimated using the mixture law concept. However, further investigation is needed to evaluate the applicability of this concept to MREs and to better understand the underlying mechanisms. When exposed to a magnetic flux density of 300 mT, the storage modulus further increased to 0.49 MPa, 0.68 MPa, and 1.13 MPa for 60, 70, and 80 wt.% CIP, respectively. At a higher magnetic field of 600 mT, the modulus rose even more significantly to 0.60 MPa, 0.88 MPa, and 1.54 MPa, respectively. These results clearly demonstrate that both magnetic field strength and particle concentration synergistically contribute to the modulus enhancement, confirming the foam’s tunable stiffness.

Muhazeli et al. [49,66] reported that incorporating CIPs shortens the LVE region of PU samples due to the formation of a matrix–filler interaction network, likely due to Van der Waals forces between the matrix and filler. Also, it was deduced that the compact distribution of CIPs causes the samples to be stiffer. They also found that higher CIP concentrations enhance the MR foam’s energy storage capacity within the elastic region, particularly at strains up to 0.0004%.

Yang et al. [71] fabricated 3D microporous MR foam based on a PU matrix with 40–70 wt.% CIP using a one-step method. Their results showed that the sample with 60 wt.% CIPs had the best overall performance, achieving a magneto-induced compression modulus of 10 MPa at 400 mT, a 65% MR effect, and a maximum magneto-induced storage modulus of 1.2 MPa. The loss modulus of 0.25 MPa also indicated high energy dissipation capability.

**Figure 5:**
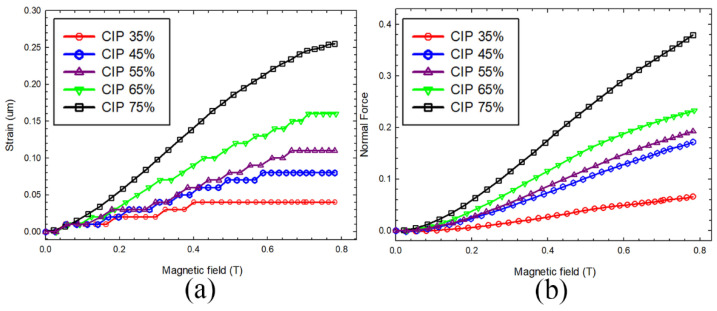
Rheological properties of MR foam in terms of magnetostrictive effect, measured by (**a**) strain versus the magnetic field and (**b**) normal force versus the magnetic field, for various CIPs contents. This figure is reproduced from reference [30] with permission from John Wiley and Sons.

Figure 5 [30] presents the experimental results of magnetostrictive effect testing in an MR foam within a PU/CIP system, where strain and normal force are plotted against the magnetic field. Figure 5a shows that, at each CIP concentration, strain increases with the applied magnetic field. As the CIP concentration rises, the magnetic moment strengthens, enhancing the interaction between magnetic particles. Notably, the MR foam with 75% CIP exhibited a significant strain enhancement due to the relative mass effect, which intensified the contact between CIPs and the foam’s microstructure. Meanwhile, Figure 5b illustrates the relationship between the normal force and the magnetic field. All MR foams demonstrated an increase in normal force with increasing magnetic field strength. Also, increasing CIP concentrations substantially results in higher normal force values. It is also noted that as the wt.% CIP increases, the saturation of strain and normal force occurs at higher values of the applied magnetic field. For instance, at 35% CIP, the induced strain is saturated at 4% under applied magnetic flux density of nearly 0.4 T, while at 65% CIP, the induced strain substantially increases and becomes saturated at around 15% under applied magnetic flux density of 0.7 T.

Padalka et al. [140] showed that higher particle permeability and saturation magnetization lead to a greater difference in the magnitude of particle–particle interaction under application of an applied magnetic field. Consequently, the largest MR effect was observed for iron (Fe), decreasing progressively, with nickel (Ni) exhibiting the smallest effect.

Winger et al. [141] investigated the effect of magnetic particle size on Young’s modulus and the MR effect in an MRE. Their results showed that increasing the particle size fraction from 20–40 µm to 80–100 µm led to a decrease in Young’s modulus from 56 kPa to 46 kPa in the absence of a magnetic field. However, under a 225 mT magnetic field, Young’s modulus did not show a significant change. Interestingly, the relative MR effect—defined in terms of Young’s modulus—increased from 30% to 55% as the particle size fraction increased from 20–40 µm to 80–100 µm under the same magnetic field, indicating improved field responsiveness with larger particles.

Wu et al. [51] studied the effect of CIP sizes (1.7, 2.8, 3.9, 4.6, and 7.2 µm) on the microstructure, mechanical properties, and magnetorheological (MR) effect of anisotropic MREs with a silicone rubber matrix. They reported that the relationship between particle size and the MR effect is not a simple linear correlation; instead, the MR effect tends to first strengthen and then weaken as particle size increases, indicating the existence of an optimal particle size. It was found that the MR effect was the most significant at an optimal particle size of 4.6 µm, although a specific mechanism for this optimal size was not presented. Based on compression tests, they observed that under the same magnetic field and strain, the stress of MREs decreased with the increasing particle size. This was attributed to the improved orientation and distribution of smaller particles within the rubber matrix. Furthermore, they reported that an increase in particle size leads to a decrease in the Young’s modulus of MRE, primarily because a larger particle size results in a reduced number of particles within the same MRE volume. Consequently, the contact area between the magnetic particles and the matrix is diminished, and the interaction between the particle surface and the matrix is weakened, leading to a decrease in the overall stiffness of the MRE. Additionally, the increased particle spacing due to a reduced particle count weakens their reinforcement effect, which may also contribute to the decrease in material stiffness.

Tagliabue et al. [142] investigated the effect of SiO_2_ and phosphate coating of CIPs on the saturation magnetization and the relative MR effect of MRE composites, specifically in terms of shear modulus. Three different commercial CIPs—without surface treatment, with SiO_2_ surface treatment, and with SiO_2_-phosphate surface treatment—were used as magnetoactive fillers. They also studied the effect of CIP concentration on the MR effect. All tests were performed under an applied magnetic field of 0.54 T. Their findings indicated that the MR effect increased with increasing CIP concentration up to 40 vol.%, but a significant drop in the MR effect was observed above 50 vol.% of filler. This behavior was explained by the theory of critical particle volume concentration (CPVC), above which the linearity approximation, as described by Jolly et al. [88], is no longer valid. The CPVC is defined as the point at which there is insufficient matrix present to fill all the space between the particles. The surface treatment of the CIPs resulted in a higher magnetization saturation of MREs with a filler content of 30 vol.%. The highest MR effect was obtained with a SiO_2_ coating. The authors proposed that the higher static and dynamic MR effect observed with the SiO_2_ coating was due to two phenomena: (1) SiO_2_-coated particles exhibit higher saturation magnetization, which leads to a higher MR effect; and (2) higher matrix–filler affinity reduces the initial shear modulus, positively affecting the MR effect.

### 7.3. Effect of Magnetic Particles on Matrix Structure

Gong et al. [13] studied the effects of incorporating magnetic particles on the matrix structure. They reported that incorporating CIPs reduces the pore size. The average pore sizes were 0.54, 0.33, and 0.23 mm for samples with 60, 70, and 80 wt.% iron content, respectively. The reduction in the pore size is likely due to the increased number of nucleation points and reduced coalescence of the cellular structure with the increasing CIP content. Schuman et al. [143] also reported an increase in the number of pores, leading to increased porosity, with the increasing CIP content. Gong et al. [13] also observed a transition from open-cell to closed-cell structures at higher CIP loadings. This is attributed to the increased viscosity of the mixture, which hinders the diffusion of CO_2_ generated during the reaction of isocyanate with water. When a magnetic field is applied, the aligned magnetic particle chains can compress the cells, causing partial degradation of the cell walls. This may promote coalescence, leading to smaller cell sizes and increased density.

**Figure 6:**
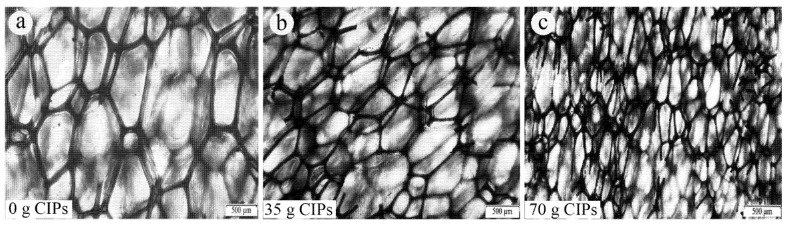
Fluorescence micrographs of MR foam samples with (**a**) 0 g CIP, (**b**) 35 g CIP, and (**c**) 70 g CIP. This figure is reproduced from reference [49] with permission from Springer Nature, Copyright 2019.

Figure 6 shows fluorescence micrographs of a PU/CIP system, illustrating the reduction in the pore size of MR foams with increasing CIP content. The average pore sizes decreased from 3.8 mm (0 g CIP, image a) to 2.9 mm (35 g CIP, image b) and 1.3 mm (70 g CIP, image c). This demonstrates that CIPs significantly influence the cell nucleation and growth process. The presence of dark regions at the foam boundaries is believed to indicate the distribution of CIPs, suggesting that they were incorporated into the struts of the MR foams during pore formation via the in situ fabrication method.

Norhaniza et al. [69] also studied the effect of adding magnetic particles on the MR foam structure. They observed that cell edges in the MR foam became sharper compared to pure PU foam. They concluded that increasing CIP content led to (i) an increase in the number of pores, (ii) more coalescence, and (iii) an increase in the number of voids between particles, resulting in higher porosity. Muhazeli et al. [49] reported a reduction in the pore area and perimeter with the increasing CIP content, which they attributed to increased melt viscosity and the CIPs acting as nucleating agents. Higher CIP content increases melt viscosity, providing greater resistance to pore growth. The smaller pores observed with higher CIP content contributed to an increased MR effect (up to 35%). The CIPs act as boundaries or barriers, influencing pore growth and limiting overall foam expansion during the foaming process.

Norhaniza et al. [69] reported a decrease in foaming time from 270 s to 188 s with the addition of 35 wt.% CIPs to PU, indicating a faster reaction rate. The presence of CIPs increases the shape factor, related to the contact angle between the PU matrix and CIPs, and reduces the energy barrier for nucleation. However, increasing the CIP concentration to 75 wt.% increased the foaming time to 291 s. This longer rising time is likely due to the rapid increase in nucleation bubbles, which reduces internal pressure and prolongs the time required for the bubbles to reach equilibrium.

Konig et al. [144] noted foam structure collapse at high filler concentrations and particle sizes larger than 100 µm. High filler concentrations increase the viscosity of the polyol and the heating capacity of the foam, reducing CO_2_ expansion and leading to foam collapse.

### 7.4. Non-Magnetic Particles

**Figure 7:**
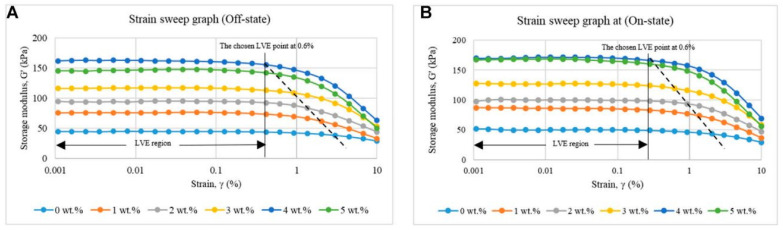
The change in storage modulus of MR foams with different concentrations of silica nanoparticles during (**A**) off-state (0 T) and (**B**) on-state (0.73 T) conditions. This figure is reproduced from reference [45] under Creative Commons Attribution License (CC BY), Copyright 2022.

Khaidir et al. [45,46] reported that adding silica nanoparticles, particularly at a 4 wt.% concentration, substantially enhanced the storage modulus of MR foam by 260%, increasing its stiffness from 45 to 162 kPa (Figure 7). This enhancement is likely attributed to the silica nanoparticles acting as crosslinkers, strengthening the filler–matrix bonding through the formation of silane coupling bonds between the silica, the PU matrix, and the CIPs. However, when the concentration of silica nanoparticles was increased to 5 wt.%, the storage modulus was noted to be lower than at 4 wt.% concentration, dropping from 162 kPa (4 wt.%) to 145 kPa (5 wt.%), particularly in the off-state condition. This reduction can be attributed to the agglomeration of silica nanoparticles in the polymer matrix when silica was added excessively. Generally, silica is known as an inorganic material that acts as a nucleating agent for polymer-matrix materials, and with every addition or increase in its composition, it typically enhances the crystallinity of the polymer composite via the interparticle connections that build between the silica particles and matrix polymer by silane coupling agent [62]. Hence, when excessive silica nanoparticles were added, the crystallinity of the material increased, which might have led to the formation of particle aggregates in the foam matrix phase. This resulted in an imbalanced distribution of silica nanoparticles that affected the dimensional stability of the foam’s cellular structure and finally caused a reverse effect of stiffness enhancement in the MR foam. Therefore, the storage modulus of MR foam with 5 wt.% silica nanoparticles decreased, indicating the excessiveness of the additive added beyond 4 wt.%.

The change in storage modulus under the influence of a magnetic field (0–0.73 T) showed a small increment (approximately 1 kPa) for each silica nanoparticle concentration due to the non-magnetic nature of silica. Micrographs revealed large open-cell pores in the MR foam, while the MR foam with silica nanoparticles exhibited more closed-cell pores, correlating with the enhanced storage modulus. This structural transition is fundamentally influenced by the presence of silica nanoparticles, which play a crucial role in the competitive processes of cell nucleation, growth, and distribution that ultimately determine the foam structure [145]. As an additive, silica nanoparticles likely act as additional nucleating sites, promoting the formation of a greater number of smaller cells [45]. Furthermore, their inherent adhesiveness significantly improves the interactions between the CIPs and the polymer matrix, reinforcing and strengthening the microstructure. This enhanced matrix–filler bonding, primarily through the formation of silane coupling bonds, contributes to the stability of the cell walls, thereby favoring the formation of a closed-cell pore structure over the larger, interconnected open-cell network. This refined cellular architecture, characterized by smaller, more defined closed cells, is directly linked to the observed enhancement in the storage modulus. It is also noted that increasing wt.% of silica nanoparticles reduces the length of the linear viscoelastic region irrespective of the applied magnetic field.

Saiz-Arroyo et al. [62] investigated the influence of nanosilica content (ranging from 1 wt.% to 9 wt.%) on the thermal and mechanical properties of both foamed and unfoamed composites. Their study demonstrated that incorporating silica nanoparticles significantly alters the structure and properties of these materials. Specifically, the addition of 1 wt.% silica nanoparticles resulted in a reduction in cell size (from 65 to 45 µm) and an increase in cell density (from 43 to 73 µm), attributed to the nucleating effect of silica in the foamed composites. Additionally, a significant enhancement in melt strength and thermal stability was noted, with the thermal stability of the foam increasing from 479 °C to 493 °C upon the incorporation of 9 wt.% silica. They also reported a 51% increase in the storage modulus of the low-density polyethylene foam with the addition of 3 wt.% silica nanoparticles. The second key finding of this research was that the improvement in mechanical properties was considerably more pronounced in foams compared to unfoamed composites. This synergistic effect, achieved by combining nanoparticles with gas dissolution foaming, is attributed to the multifunctional role of the nanoparticles, which influence both the polymeric matrix morphology and the cellular structure of the foams.

Saiz-Arroyo et al. [62] also reported a 51% increase in the storage modulus of low-density polyethylene foam with the addition of 3 wt.% silica nanoparticles. The LVE region decreased upon the addition of silica nanoparticles, indicating a more rigid and stiffer structure, further enhanced under a magnetic field (on-state condition). This stiffening effect is attributed to the adhesiveness of the silica nanoparticles [46]. The pure MR foam exhibited a slightly longer LVE region in the off-state condition. All MR foams showed shorter LVE regions in the on-state condition compared to the off-state.

In addition to influencing the MR properties, non-magnetic particles also affect the foam structure. According to the study by Saiz-Arroyo et al. [62] on the polyethylene/silica nanocomposite system, silica nanoparticles act as nucleating agents, promoting the formation of silane coupling bonds through hydrogen bonding, which enhances the adhesion between the particles and the matrix. The large surface area of nano-sized silica particles intensifies bubble nucleation and allows for the formation of more silane coupling bonds. Silica increases the crystallinity of the polymer composite through interparticle connections between the silica particles and the matrix polymer via the silane coupling agent. However, excessive silica nanoparticle addition can increase crystallinity, potentially leading to particle aggregation and an uneven distribution of silica nanoparticles. This can negatively impact the dimensional stability of the foam’s cellular structure and counteract the stiffness enhancement. MR foams with silica nanoparticles tend to have more closed-cell pores compared to the more open-cell structure of typical flexible foams.

### 7.5. Orientation of Magnetic Particles

Gong et al. [13] reported that CIP orientation significantly improves compressive stress along the aligned direction in anisotropic MR foams. For example, the compressive strength of an anisotropic sample along the magnetic chain direction reached 1053.5 kPa at 3.9% strain, which was 57 times higher than that in the perpendicular direction (18.5 kPa) and 878 times higher than that of the blank unfilled PU foam (1.2 kPa). The zero-field modulus of anisotropic samples was also higher than that of isotropic samples with the same iron content, likely due to the fact that CIP orientation reduces interparticle distance, enhancing magnetic particle interaction under a magnetic field.

Muhazeli et al. [146] investigated the effect of curing conditions on MR foam composed of CIPs within a PU foam matrix. MR foams with 35 wt.% CIPs were fabricated via an in situ method under two different curing conditions: in the absence and presence (magnetic field of 0.2 A) of a magnetic field, referred to as isotropic and anisotropic conditions, respectively. The results demonstrated an oriented structure of CIPs between the struts of the MR foam after curing under anisotropic conditions. Rheological testing revealed that the anisotropic MR foam exhibited the highest relative MR effect in view of shear modulus (under a 0.8 T magnetic field), approximately 89% (from 3.00 to 5.68 MPa), compared to the isotropic MR foam, which possessed only 10% (from 5.13 to 5.67 MPa). It was observed that the range of storage modulus values for the anisotropic MR foam was higher compared to the isotropic MR foam, even though the isotropic MR foam sample was notably stiffer in the off-state. The increment of storage modulus across the swept frequency range in both the on-state and off-state also differed slightly for each curing condition, revealing that the anisotropic MR foam achieved the highest storage modulus, approximately 1.33 MPa, compared to the isotropic MR foam and the virgin foam, which presented values of approximately 1.14 MPa and 0.84 MPa, respectively. This result is consistent with findings from their previous studies where the storage modulus increased with an increase in the current applied to the MR samples [13]. Although the presence of the magnetic field during the curing of MR foam is mentioned as the primary reason for the observed changes in the MR behavior of the anisotropic sample, no definitive mechanism was presented for these changes. However, it appears that alterations in the configuration of the particles as a result of the applied magnetic field are the main contributing factor.

Yang et al. [71] also investigated the effect of CIP content and particle aggregation on the magneto-mechanical properties of MR foam. They reported that the compressive modulus increased with the increasing CIP content, a phenomenon attributed to the significant stiffness enhancement provided by the CIPs. It is important to note that the compression was applied parallel to the particle chain direction in these tests. Furthermore, for the same CIP content, anisotropic PU MR foam exhibited a higher storage modulus than isotropic samples, indicating that aligned particle chains have a stronger reinforcing effect compared to randomly dispersed particles.

The orientation of magnetic particles significantly affects the long-term properties of MR solids. In a study by Zhou et al. [147], the stress–strain behavior of isotropic and anisotropic MREs at different stress amplitudes under equi-biaxial cyclic loading revealed that anisotropic samples endure a greater number of failure cycles than isotropic ones. For instance, under engineering stress control at a stress amplitude of 0.75 MPa with a minimum stress of zero and in zero-field conditions without magnetic field application, anisotropic samples failed at 3252 cycles, whereas isotropic samples failed at only 561 cycles. Scanning electron microscopy (SEM) was employed to observe the fracture surface morphologies of isotropic and anisotropic MREs after failure under various loading conditions. For isotropic samples, cyclic loading induced debonding of particles from the silicone matrix, leading to the formation of voids and cavitation that ultimately resulted in failure. Other particles and their agglomerates remained on the fracture surface, and their interfaces with the silicone matrices were very distinct, indicating poor interaction. The fracture surface of anisotropic MRE samples, however, differed. While dynamic loading still induced some particle debonding, it was less prevalent compared to isotropic samples under the same loading conditions. The remaining particles in anisotropic samples were found to be robustly embedded in the silicone matrices, exhibiting an improved interface. This morphological evidence further supports the conclusion that the fatigue resistance of anisotropic MREs is superior to that of isotropic ones at equivalent loading levels. However, no mechanism was proposed for a better interface between CIPs and the matrix in anisotropic samples.

Khairi et al. [148] investigated the tensile behavior of isotropic and anisotropic MREs composed of silicone liquid and 70 wt.% CIPs, both in the presence and absence of a magnetic field. Their results showed that even without magnetic stimulation, the anisotropic MRE (with aligned CIPs) exhibited a higher tensile strength of 1.556 MPa compared to 1.524 MPa for the isotropic MRE. This increase is attributed to the chain-like alignment of CIPs parallel to the tensile load direction, which enhances load transfer efficiency. However, this improvement in strength came at the cost of lower elongation at break—127.2% for the anisotropic sample versus 165.61% for the isotropic one—indicating reduced flexibility and a shift toward more brittle behavior. This trade-off highlights how particle alignment not only strengthens the material but also restricts its ability to deform, making it less ductile. The aligned particles form rigid microstructures that resist deformation more effectively but also limit the overall stretchability of the matrix. Upon application of a magnetic field, both MRE types exhibited increased tensile strength. The isotropic sample reached 1.804 MPa with 143.30% elongation, while the anisotropic sample showed 1.643 MPa and 118.86% elongation. These results confirm that CIP alignment significantly impacts not just compressive strength but also tensile strength, flexibility, and deformation behavior, with anisotropic structures offering higher mechanical stiffness and strength at the expense of elasticity.

As demonstrated, aligned CIP structures significantly enhance compressive stress along the field-aligned direction, increase the relative MR effect, improve long-term properties, and contribute to higher compressive moduli and tensile strength, albeit at the expense of reduced elongation. However, the long-term stability of CIP alignment under operational conditions—particularly high-frequency mechanical loading, repeated magnetic cycling, or thermal fluctuations—remains insufficiently understood. Over extended use, factors such as matrix creep, interfacial debonding, or fatigue-induced particle mobility may lead to gradual disintegration of the aligned microstructure, thereby diminishing field responsiveness and mechanical performance. To ensure sustained functionality in real-world applications, future studies should focus on quantifying the durability of alignment. This could be achieved through methods such as in situ imaging, magnetic remanence analysis, or rheological fatigue testing under varying field and strain amplitudes. Such investigations are essential to bridging the gap between short-term performance gains and long-term reliability in MR foam systems.

### 7.6. Frequency and Magnetic Flux Density

In polymeric matrix MR foams, the motion of polymer molecular chains is frequency-dependent. Gong et al. [13] showed that the zero-field modulus of both isotropic and anisotropic samples increased with the increasing test frequency, which they attributed to the frequency dependence of the polymer matrix. At higher frequencies, the mobility of polymer molecular chains cannot keep pace with the external stimuli, leading to increased rigidity and a higher zero-field modulus.

Choi et al. [73] reported that the storage and loss moduli of silicone-based MR foams with CIPs as magnetic particles were only weakly frequency-dependent. However, the equivalent damping was strongly frequency-dependent. The storage modulus slightly decreased with the increasing frequency, possibly due to the Mullins effect or stress softening, a common phenomenon in particle-filled elastomeric materials [149]. While there is no single, universally accepted explanation for the Mullins effect, Bouche et al. [150] suggested that this stress softening is due to the disentanglement of polymer network chains caused by the breakdown of particle–filler–matrix interactions.

Yang et al. [71] reported that the storage modulus of PU-matrix, CIP-filled MR foam, increased with the increasing magnetic flux density, demonstrating a clear MR effect. The storage modulus also increased with the increasing shear frequency, likely because the dynamic response time of the polymer matrix decreases with the increasing frequency, leading to increased stiffness. With the exception of anisotropic MR foam samples containing 70 wt.% CIPs, the loss modulus of other samples (40–60 wt.% CIP) exhibited minimal variation with the magnetic field. The loss modulus of the 70 wt.% anisotropic samples decreased sharply with the increasing magnetic flux density. This is likely due to the high CIP content enhancing interparticle interactions. The increased binding force of the CIPs on the matrix limits the energy dissipation capacity of the matrix molecular chains.

Maranville and Ginder [151] performed a viscoelasticity analysis on MR foam produced by entraining PU foams in MR fluid suspensions (CIPs and polydimethylsiloxane or silicone oil). They measured the effects of strain amplitude, frequency, and magnetic field strength on viscoelastic properties. They reported that increasing strain level and magnetic field strength led to increasingly nonlinear viscoelastic behavior. Norhaniza et al. [152] reported a 6 KPa increase in the storage modulus of a pure MR foam with 75 wt.% CIPs when the magnetic field was increased from 0 to 0.78 T.

The work of Poojary et al. [153] presents a comprehensive examination of how excitation frequency and magnetic field strength jointly influence the dynamic mechanical behavior of isotropic MREs. The authors studied a two-component RTV silicone-based MRE with 27 vol% CIPs, subjecting the material to harmonic compression across a frequency range of 8–24 Hz and magnetic flux densities of 0, 0.1, 0.2, and 0.27 T at a fixed strain amplitude of 0.25%. Their results demonstrate that both frequency and magnetic field exert a coupled effect on the stiffness and energy dissipation characteristics of MREs. Specifically, higher frequencies lead to increased stiffness, a trend that is further amplified in the presence of a magnetic field. This synergistic behavior is evident in the progressively steeper force–displacement hysteresis loops and greater loop areas with the increasing magnetic flux density, indicating enhanced energy storage and dissipation capacity. For example, at 16 Hz and 1.5% strain, the real part of stiffness increased from 168.06 N/mm at 0 T to 196.836 N/mm at 0.27 T based on model predictions, with experimental measurements yielding comparable results (176.245 N/mm to 204.822 N/mm).

This interplay is particularly relevant to the fatigue performance of MR solids. Materials operating under cyclic loading at elevated frequencies and in magnetized states are subject to increased internal friction, hysteresis losses, and localized heating—all of which can accelerate microstructural degradation over time. The enhanced stiffness under these conditions suggests improved load-bearing capacity but may also correspond to higher mechanical stress concentrations within the matrix–filler network. Moreover, the frequency-dependent viscoelastic response, governed by the matrix’s time-dependent relaxation dynamics, interacts with the field-induced particle chaining mechanisms, resulting in a complex, nonlinear evolution of the material’s internal structure during long-term use. The modeling framework employed by Poojary et al., based on a fractional Zener model combined with a linearized Bouc–Wen element, effectively captures these multi-field dependencies. Notably, the use of a magnetic stiffness term allows for quantifying the magnetic contribution to the overall dynamic modulus, while frequency effects are incorporated via fractional derivatives reflecting rate-sensitive viscoelastic behavior. The close agreement between experimental and model-predicted dynamic stiffness (fitness values > 93%) supports the model’s suitability for performance forecasting.

### 7.7. Temperature

Temperature is one of the most significant factors influencing the performance of polymer composites. MR materials, when used as damping or transmission media, are often subjected to elevated operating temperatures, particularly in high-power MR devices. It has been reported that as the temperature increases above room temperature, the zero-field modulus of polymer matrix-based MR foams decreases [83]. This reduction is primarily due to the increased thermal energy, which enhances the mobility of the polymer molecular chains and permits slight movement of the embedded particles.

Wang et al. [154] measured the magnetic hysteresis loops of CIPs and found that elevated temperatures significantly degrade their magnetization properties. The degradation rate was found to increase approximately in sync with temperature. Experimental results showed that both the mass magnetization and coercivity of the MR particles decreased as temperature rose, with the effect being particularly pronounced at higher temperatures. The decline in mass magnetization is primarily attributed to the fact that rising temperatures accelerate atomic motion, which disrupts the alignment of magnetic moments within the particles. This disruption results in a reduction in the magnetic moment per unit mass of CIP. Specifically, as the temperature increased from 47 °C to 147 °C, the saturation mass magnetization decreased from 198.8 emu/g to 187.2 emu/g. However, a more dramatic drop of 25.8 emu/g was observed when the temperature rose from 347 °C to 447 °C. Over the full temperature range of 47 °C to 447 °C, the coercivity reduction reached approximately 90.5%. This phenomenon is mainly attributed to the thermal fluctuation of the blocking moments caused by the anisotropy energy barrier.

Wan et al. [155] investigated the viscoelastic properties of anisotropic MRE samples (composed of silicone rubber with a 30% volume fraction of iron particles) under various temperatures (25 °C to 60 °C) and uniaxial harmonic compressive loading. A transition behavior in the dynamic modulus of the anisotropic MRE samples was observed at around 50 °C. The storage modulus initially decreased with increasing temperature up to 50 °C, then either increased or remained stable as the temperature rose to 60 °C. They reported that this transition behavior around 50 °C could be attributed to the combined effects of (i) the alpha-phase transition of the particle-reinforced silicone rubber and (ii) dipole interactions between iron particles under a magnetic field when the silicone rubber matrix softened due to the elevated temperature. According to the differential scanning calorimetry results, the transition began at approximately 50 °C and reached an exothermic peak at 58.66 °C.

Hemmatian et al. [156] experimentally investigated the temperature dependence (−10 °C to 50 °C) of the viscoelastic properties of an MRE composed of silicone rubber and a 25% volume fraction of CIPs, operating in shear mode. The results revealed a significant effect of temperature on both the linear and nonlinear viscoelastic properties of the MRE, with reductions in both storage and loss moduli as temperature increased under shear strain. However, the temperature sensitivity of the MRE diminished when the shear strain exceeded the critical strain. The study also showed that the temperature effect was more pronounced at higher temperatures and excitation frequencies, while it became less significant with the increasing magnetic field strength. Interestingly, an increase in temperature enhanced the MR effect, although no clear trend could be established with respect to excitation frequency. Additionally, a phenomenological model was proposed to predict the storage and loss moduli of the MRE as functions of excitation frequency, applied magnetic flux density, and temperature. Their model demonstrated strong predictive accuracy for the MRE system (25% CIP in a silicone rubber matrix) under various conditions, with an average error of 3.2% and a coefficient of determination of 0.99 compared to experimental results.

Wen et al. [157] also investigated the effect of temperature on the magneto-mechanical properties of anisotropic MREs. Their results showed that both the initial modulus and the magnetically induced modulus decreased with increasing temperature. As the temperature increased from 20 °C to 50 °C, the initial modulus dropped from 165.0 kPa to 78.8 kPa, while the maximum magnetically induced modulus decreased from 99.5 kPa to 41.2 kPa. They concluded that this reduction in magnetically induced modulus was due to the rotation of particle chains within the elastomer matrix. A theoretical analysis of the temperature-dependent rotation led to an expression of the magnetically induced modulus as a function of both the magnetic field and the initial modulus.

As previously mentioned, in CIP-filled MR foams with a PU matrix, increasing the temperature leads to a reduction in both storage and loss moduli. However, it has been reported that adding silica nanoparticles—ranging from 0 to 11 wt.%—can mitigate this effect by strengthening the interfacial interactions between the CIPs and the PU foam matrix [83]. Additionally, the incorporation of silica nanoparticles was found to enhance the thermal stability of the MR elastomer, as evidenced by an increase in the thermal decomposition temperature from 210 °C to 235 °C. More elaboration about the effect of CIPs and silica nanoparticles on thermal stability is presented in further sections.

## 8. Functional Properties and Applications

MR foams, with their unique combination of tunable mechanical properties and responsiveness to magnetic fields, have emerged as promising materials for a wide range of functional applications. This section explores the diverse capabilities of MR foams, highlighting their potential in areas such as acoustic absorption, vibration mitigation, soft robotics, sensing, and energy harvesting. The ability to dynamically control their properties through external magnetic fields enables the development of smart devices with enhanced performance and adaptability. Additionally, how the inherent characteristics of MR foams—such as porosity, matrix composition, and magnetic filler distribution—can be modified to achieve desired functionalities is examined. This section provides insights into the practical significance of MR foams and their future prospects.

### 8.1. Acoustic Absorption Characteristics

MR foams, with their high elasticity, active real-time response to external stimuli, excellent vibration damping, and stability in ambient conditions, are considered promising candidates for smart sound and acoustic absorption applications [70]. The concept of using MR foams for active acoustic absorption was first proposed by Scarpa et al. [35,36], who studied PU MR foam fabricated ex situ by immersing open-cell PU foam in MR fluid. They found that the composite foam exhibited a high acoustic absorption coefficient in the 1000–2800 Hz frequency range. The MR foam also demonstrated the ability to shift the peak acoustic absorption coefficient within a given frequency bandwidth by applying constant intensity magnetic fields. Using two different permanent magnets (neodymium at 1.25 T and ceramic at 0.25 T), they observed a shift in the sound absorption frequency from 1.8 to 2.2 kHz. This ~400 Hz shift to a higher frequency range suggested the potential use of auxetic MR foam as a medium in noise control applications.

Zielinski et al. [38] investigated the tunable acoustic absorption of ex situ-fabricated MR foam, focusing on its potential as a semi-active sound absorber. They compared conventional foam with a positive Poisson’s ratio, saturated with MR fluid, in both homogeneous (single-porosity) and non-homogeneous (dual-porosity, with larger, irregular, mesoscopic pores within a microporous domain) configurations. Their findings revealed that dual-porosity MR foam exhibited superior acoustic absorption performance compared to single-porosity MR foam. Notably, the peaks in the absorption curves of dual-porosity MR foams showed a significant shift (approximately 400–500 Hz) under the influence of a magnetic field, relative to field-free conditions or clean foams. This frequency shift resulted in enhanced acoustic absorption at higher frequencies, demonstrating the suitability of non-homogeneous porosity for magnetically controlled noise absorption.

In a study conducted by Muhazeli et al. [49], the influence of CIP content on the acoustic absorption properties of MR foams was examined. The authors reported that increasing the CIP content significantly enhanced sound absorption performance. Specifically, as the CIP content increased from 0 to 35 g and 70 g, the peak sound absorption coefficient (SAC) shifted from 5544 Hz to 3744 Hz and 2568 Hz, respectively. These results demonstrate the potential of using MR foams in systems subjected to specific frequency excitations. It was also observed that the SAC increased across the entire frequency range with higher CIP content, consistent with observations reported in other studies [158,159]. According to the authors, this behavior can be attributed primarily to the combined effects of pore structure and CIP concentration. Larger pores were said to allow sound waves to pass through with minimal diffraction, whereas smaller pores caused more diffraction, leading to greater surface friction and energy dissipation in the form of heat. Additionally, the study noted that CIPs contributed to acoustic energy dissipation by both absorbing vibrational energy and acting as reflectors that induce destructive interference. As a result, higher CIP content was associated with greater sound reflection and enhanced overall energy loss.

Muhazeli et al. [70] investigated the effect of a magnetic field on the sound absorption of MR foam. They reported that applying a magnetic field using permanent magnets (0.25 T and 0.5 T) shifted the frequency range of the MR foam, indicating its potential as a semi-active material for adaptive sound-controllable devices. In the off-state condition, there was no change in the elastic parameters or pore size. The addition of CIPs and the application of a magnetic field contributed to the effectiveness of the MR foam as a semi-active material with variable viscous damping and stiffness properties. While applying a magnetic field decreased the sound absorption coefficient values, the substantial shift in peak frequency from the middle to a higher range (the “shifting effect”) allowed for tuning of the sound absorption characteristics.

### 8.2. Adaptive Vibration Mitigation and Damping Effect

The three main approaches to vibration mitigation are active, passive, and semi-active [160]. Semi-active devices have gained significant attention because they consume less power and offer better fail-safe operation than active systems while being more adaptable than their passive counterparts [161]. In recent years, semi-active vibration control devices based on smart materials have attracted increasing interest [162,163].

The damping effect in MR materials stems from their ability to resist deformation or flow by increasing energy dissipation when subjected to an applied magnetic field. Carlson et al. [164] described the basic elements of a simple, linear MR foam damper as an early concept for using MR foam in an application requiring no seals or bearings and using only about 3 mL of MR fluid. Damping in MR materials relies on several mechanisms. The most widely accepted mechanism is the formation of chain-like particle structures aligned along magnetic flux lines. These structures form when discrete magnetic particles, typically suspended in a fluid or embedded in an elastomer matrix, become magnetized upon the application of an external magnetic field. The induced magnetic dipoles on adjacent particles lead to attractive forces, causing them to align end-to-end and form continuous or semi-continuous chains parallel to the magnetic field direction [102]. This rearrangement fundamentally alters the material’s resistance to motion. In their role in damping, these chain-like structures significantly enhance the material’s yield stress and dynamic modulus. This demands more force for deformation as the chains resist disruption. As the material deforms, these reconfigured particle chains undergo internal friction and viscous losses as particles attempt to move relative to each other or the surrounding matrix, thereby dissipating mechanical energy as heat. This improved energy dissipation is the essence of the damping effect. While their characteristics depend on the surrounding matrix (e.g., in a solid elastomer, chains are relatively fixed; in a fluid, they can rearrange more freely), the principle of energy absorption through structured resistance holds. In MR fluids, where particles have greater mobility, the constant rearrangement and breaking/reforming of chains, along with interparticle interactions, contribute to energy loss through viscous friction as the fluid flows [165]. In MR elastomers, Chen et al. and Fan et al. [166,167] indicated that damping properties strongly depend on interfacial slipping between the particles and the matrix. In MR foams, the presence of bubbles significantly influences damping by introducing additional energy dissipation mechanisms. The compressibility and deformation of bubbles add viscoelastic behavior, while stress redistribution around the voids enhances interfacial slipping and friction between particles and the matrix. The disruption of continuous particle chain alignment by bubbles increases localized friction, and air drag during bubble deformation contributes to further energy dissipation. These combined effects make MR foams highly tunable for damping applications, with properties that can be properly tailored by adjusting bubble size, distribution, and matrix composition.

### 8.3. Soft Robotics and Biomedical Applications

Recent developments in robotics have focused on incorporating materials with adjustable stiffness rather than relying on passive materials, particularly in applications such as soft robotic grippers for improved object grasping and handling [168]. This has generated interest in the characteristics and behavior of MR foam as a new soft, smart material with adjustable properties responsive to applied magnetic fields.

Park et al. [169] proposed a controllable tactile device capable of generating repulsive forces from soft human tissues and verified its effectiveness experimentally. The device was fabricated by immersing porous PU foam in MR fluid and encapsulating it with adhesive tape. They measured and analyzed the repulsive force and relaxation stress as a function of magnetic field intensity to verify the device’s suitability for soft human tissues like the liver. Psychophysical testing further validated the device’s effectiveness and practical applicability. Specifically, the psychophysical test, conducted with 20 volunteers, evaluated their ability to differentiate between the repulsive forces mimicking various human tissues (muscle: 0.22 N, heart: 0.37 N, intestine: 0.68 N) after short training periods (15 or 30 min). The results demonstrated that participants could accurately recognize these different repulsive forces, with survey responses showing high agreement that the proposed sample could realize the forces for each organ (mean scores of 4.25 and 4.10 out of 5 for recognition and realization, respectively). This confirmed the device’s practical applicability by showing that users can easily distinguish between different tissue stiffnesses with minimal training. They subsequently developed a new type of tactile device called MRTTC (MR materials-based tactile transfer cell) for robot-assisted minimally invasive surgery (RMIS) and evaluated its field-dependent repulsive force performance [170]. The MRTTC consists of a 1 mm thick MRE cover wrapping PU foam and an MR fluid core absorbed by the PU foam. The field-dependent repulsive force generated by the MRTTC replicates the stress relaxation behavior of human organs, effectively accounting for the nonlinear viscoelastic phenomena of MR materials. The similarity between the measured and calculated Young’s moduli of human organs and the device suggests that the MRTTC can be effectively used in RMIS, allowing surgeons to feel the same as the target organ. These advancements by Park et al. [169] represent a significant stride toward integrating realistic haptic feedback into robotic systems, especially for delicate procedures like robot-assisted minimally invasive surgery. By precisely replicating the viscoelastic properties and stress relaxation behavior of human tissues, MR-based tactile devices like the MRTTC can fundamentally enhance a surgeon’s dexterity and decision-making by providing a critical sense of touch. This capability is not merely an incremental improvement but a transformative development that could lead to safer, more precise, and, ultimately, more effective surgical outcomes. Beyond surgery, the underlying principles of controllable tactile rendering using MR materials hold immense promise for other areas of robotics, including advanced prosthetics, realistic haptic interfaces for training simulations, and nuanced human–robot interaction in collaborative environments, paving the way for more intuitive and adaptable robotic technologies across various industries.

One of the most compelling applications of MR foams in soft robotics and biomedical fields is drug delivery. Due to their stimuli-responsive drug release behavior, inherent reservoir structure for drug storage, and the ability to achieve rapid, real-time control of drug release through magnetic field-triggered large deformations, MR foams are among the most promising candidates for this application. Drug delivery systems based on porous, soft biomaterials can release loaded drugs at the target site under external stimuli without causing mechanical damage to the human body. Sun et al. [171] proposed a soft capsule composed of a hard magnetic elastomer foam with excellent biocompatibility and significant deformability for magnetically controlled, on-demand drug delivery. The MR foam was fabricated using NdFeB microparticles (5 μm) as the magnetic filler, Ecoflex as the matrix, and sugar as the template. The mixture was cast into molds and baked on a hot plate. After solidification, the magnetic mixture was removed from the molds and rinsed in water to dissolve the sugar, rendering the foam porous and flexible. Finally, the porous samples were placed in a magnetic field to directionally magnetize the dispersed hard magnetic particles. The fabricated MR foam capsule demonstrated an adjustable drug release rate ranging from 0.02 to 1.7 mL/min. Its deformation-triggered drug release profile under a magnetic field was accurately predicted, enabling approximately 85% accuracy in drug dosage regulation and over 90% maximum cumulative drug release. The MR foam capsule also proved capable of acting as a soft robot for magnetically driven drug delivery in a human stomach model. These advancements by Sun et al. showcase the transformative potential of MR foam-based systems for intelligent, on-demand drug delivery. By enabling precise dosage control and targeted delivery within complex biological environments, such soft capsules can revolutionize personalized medicine, minimize side effects, and improve therapeutic outcomes. This research paves the way for a new generation of magnetically actuated biomedical robots and smart implantable devices, fundamentally altering future treatment methodologies across diverse medical applications.

**Figure 8:**
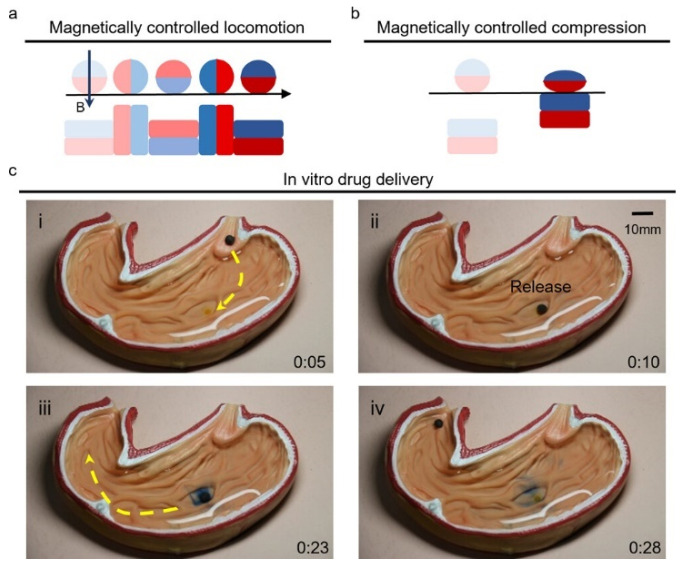
Drug delivery of the MR foam capsule in a human stomach model. Illustrations of the (**a**) locomotion and (**b**) compression of the MR foam capsule under the magnetic field. (**c**) In vitro drug delivery of the MR foam capsule in a model of the human stomach (yellow dashed arrow indicates path). The time stamps (in minute:second format) indicate the elapsed time during the experiment. Reprinted (adapted) with permission from [171]. Copyright 2023 American Chemical Society.

Figure 8 illustrates the in vitro drug delivery performance of the MR foam capsule developed by Sun et al. [171], tested within a human stomach model. Leveraging the hard magnetic properties of the embedded particles, the capsule could be remotely actuated using an external magnetic field, enabling controlled navigation and deformation in confined environments. As shown in Figure 8a, the rotation of an external magnet induced rolling motion of the capsule along the surface. By adjusting the distance between the magnet and the capsule, compressive deformation could be controlled, as demonstrated in Figure 8b. To simulate drug release, blue ink was used as a model drug and was pre-loaded into the porous structure of the foam under vacuum conditions. The capsule was then magnetically guided into the stomach model through the cardia. Due to its flexibility and soft structure, it was able to traverse irregular internal surfaces. Upon reaching the target site, the magnetic field strength was increased to approximately 240 mT by bringing the magnet closer to the capsule (Figure 8c-ii), which triggered compression and drug release through the foam’s pores (Figure 8c-iii). The dosage could be modulated by adjusting the strength and cycling of the magnetic field. Following the release, the capsule exited the stomach by rolling toward the pylorus (Figure 8c-iv). These results highlight the potential of MR foam capsules for future in vivo drug delivery applications, particularly in soft organ systems such as the stomach.

### 8.4. Sensors

There is a constant demand for soft sensors that innately possess desirable features such as high durability, flexibility, and mechanical resilience. Among the various smart materials developed to date, MR solids—capable of exhibiting magnetostriction in the presence of a magnetic field—hold strong potential for implementation in soft sensors due to their ability to adapt to multi-scale and dynamic deformations while maintaining mechanical compliance.

To date, many studies have focused on low-modulus MREs (less than 100 kPa), which can limit their potential application in sensors due to their short lifespan and low durability. Tasin et al. [172] investigated MREs with a storage modulus above 300 kPa to enhance both magnetostriction magnitude and reaction force (normal force). MREs with 60, 70, and 80 wt.% of CIP were prepared, and it was shown that both the magnetostriction percentage and normal force increased with the CIP concentration. The highest magnetostriction magnitude of 0.075% was obtained with 80 wt.% CIP. The mid-range modulus MREs developed in their work can robustly achieve the required magnetostriction response and hold promise for the development of next-generation sensor technologies.

A limitation of MR materials for sensor applications has been the need for a strong magnetic field (1 T) and low sensitivity at lower fields. Norhaniza et al. [139] addressed this by fabricating a PU-based MR foam designed to achieve strain at magnetic fields below 1 T. Using an in situ fabrication method with varying CIP compositions, they found that a 75 wt.% CIP MR foam exhibited the highest strain (1.66%) at 0.45 T and a sensitivity of 0.0146%/mT. They suggested that the low density, large pores, and long struts of this MR foam contributed to its high flexibility and elongation, enabling high strain percentages within a practical magnetic field range. This MR foam shows promise for sensor applications.

Qi et al. [173] designed a self-powered magnetic-field sensor based on an MRE film and a triboelectric nanogenerator, capable of sensing both time-varying and uniform magnetic fields. This sensor generates an electrical signal in response to magnetically induced deformation of the MRE film, without requiring an external power supply. It exhibits a fast response time (20 ms) and good magnetic-field sensing performance. In this application, magnetostriction is the critical parameter of the MRE film. The designed self-powered magnetic-field sensor with 60 wt.% MRE showed a maximum sensitivity of 16 mV/mT for magnetic fields ranging from 40 to 100 mT. The results also demonstrated that the sensitivity of the sensor could be adjusted by tuning the MRE composition and device parameters.

### 8.5. Energy Harvesting

Energy conversion is a significant challenge in the development of multifunctional materials. Generally speaking, ‘energy conversion’ encompasses both actuation, where electrical energy is used to produce mechanical displacement, and sensing and energy harvesting, where mechanical energy is harnessed to generate electricity (for instance, from vibrations or periodic motions). MR solids have been demonstrated to be effective materials for converting mechanical energy to magnetic energy and, subsequently, to electrical energy. By definition, the magneto-mechanical coupling that describes the dependence of the magnetic induction field on mechanical stress is called the Villari effect, also known as magnetoelastic coupling or inverse magnetostrictive coupling [174].

MR solids, when placed in the air gap of an electromagnet that applies a controlled, constant static magnetic flux density and subjected to a varying mechanical shear strain, undergo relative displacement of the embedded particles. This displacement modifies interparticle interactions, which, in turn, alter the shear modulus of the MR solid and cause a change in permeability under a magnetic field. This change in permeability, along with the applied magnetic field, induces a magnetic flux density across the entire magnetic circuit. This change in magnetic flux is then converted into an electrical signal via a sensing or ‘search’ coil.

Sebald et al. [174] focused on the capacity of CIP/silicone rubber MR elastomer to convert mechanical energy into electrical energy through magneto-mechanical mechanisms. In their study, since an external magnetic field is used to achieve this mechanical-to-magnetic domain coupling, it is termed the pseudo-Villari effect. They developed an experimental setup to characterize the dependence of magnetic properties on shear strain, achieving variations of up to ΔB ≈ 10 mT for a strain of 50% and a bias magnetic flux density *B*_0_ ≈ 0.3 T. Isotropic soft and hard elastomers exhibited different behaviors, with soft elastomers showing substantial pseudo-Villari effects, while hard elastomers did not. Interestingly, anisotropic soft and hard elastomers showed rather similar behavior regarding the pseudo-Villari effect (ΔB = 10.2 mT and 9.4 mT, respectively, for 50% strain and a bias magnetic field of 0.3 T). Based on this observation, they concluded that the pseudo-Villari effects in anisotropic MREs depend solely on the particle type and volume fraction, likely because they originate from dipole–dipole and induction field–dipole interactions. The developed experimental setup was further used to validate a proposed model, which can be used to determine the global composite relative permeability (μ_MRE_) [175].

Diguet et al. [176] investigated the effect of different magnetic particle volume fractions on the energy conversion characteristics of CIP/silicone rubber MREs. Their work demonstrates that the output voltage depends on (i) the magnetic particle volume fraction, (ii) the shear amplitude, and (iii) the applied constant magnetic field. While the results revealed that the voltage amplitude increases with applied shear, the effects of magnetic particle volume fraction and applied bias field were less straightforward. It was found that increasing the magnetic particle volume fraction beyond 15% did not significantly improve the voltage output, and that a maximum voltage was achieved at a specific optimal applied field. The best condition for voltage amplitude was observed with a 0.2 T applied magnetic field and a 50% applied shear amplitude, using different magnetic particle volume fractions. They also reported that, as both the magnetic particle volume fraction and the applied constant field seem to have a limited impact on ΔB, the most effective way to improve Δ*B* is to increase the applied shear strain deformation.

In this context, MR foams emerge as a promising alternative for energy harvesting applications due to their distinct advantages over other MR materials. Specifically, MR foams can achieve significantly larger deformations more easily, especially when utilizing a porous matrix like PU foam instead of traditional silicone rubber found in conventional MREs. This enhanced deformability is crucial for energy harvesting, as the change in shear-induced magnetic flux density, a key factor for energy generation, has been found to be weakly dependent on the matrix choice in MREs [174]. Therefore, the inherent high deformability of MR foams allows for a much stronger compression-dependent magnetic permeability. This robust coupling between their magnetic and mechanical properties ultimately enables enhanced energy conversion and opens new and broader application possibilities.

Among the various strategies under exploration, several materials and processes stand out as particularly promising yet still face critical shortcomings that require targeted research. On the material side, low-modulus, thermally stable elastomers, such as silicone and fluorinated polyurethanes, offer an attractive flexibility, but they often suffer from low thermal resistance, high processing viscosity, or cost-related scalability issues. Similarly, surface-engineered magnetic particles with coatings such as silane and dopamine have demonstrated improved interfacial bonding, yet challenges persist in achieving uniform coating thickness and maintaining long-term chemical and mechanical stability. From a processing standpoint, in situ magnetic field-assisted curing remains a high-impact technique for inducing anisotropy, though it demands precise control of curing kinetics and field distribution. Meanwhile, constrained foaming methods show potential in achieving uniform cellular architectures, but the lack of standardized protocols and the risk of pore collapse or density gradients limit their current reproducibility. Addressing these material- and process-specific shortcomings will be crucial for transitioning MR foams from lab-scale demonstrations to robust, scalable, and application-ready smart material systems.

## 9. Challenges and Future Directions

While MR foams have demonstrated significant promise in a variety of applications—from vibration-damping systems to soft robotics and smart biomedical devices—several challenges remain that limit their broader implementation. This section explores key limitations in current MR foam technology and outlines future research directions aimed at overcoming these obstacles. Specifically, we discuss issues related to thermal stability and conductivity, the critical role of matrix–particle interface control, and the complexities of accurately regulating bubble formation during fabrication. By identifying and analyzing these targeted challenges, we aim to provide a focused roadmap for future research and development efforts—one that directly supports the design of MR foams with improved thermal management, enhanced interfacial bonding, and precisely engineered microstructures for reliable and application-specific performance.

### 9.1. Thermal Stability and Conductivity

A critical feature of MR foams that influences their applications is their thermal stability. MR foams are susceptible to degradation, which can lead to a reduction in the storage modulus when exposed to thermal stress from the operating system of a device [46]. The working temperature range of polymeric MR foams typically spans from −40 °C to 150 °C, limited by the thermal stability of the polymer matrix. PU-based foams are typically limited to around 120 °C, while silicone-based foams can withstand temperatures up to 200 °C. This superior thermal resistance of silicone-based foams (polysiloxanes) is primarily attributed to the inherent strength and flexibility of their inorganic silicon–oxygen (Si-O) backbone, which possesses higher bond energy and greater oxidative stability compared to the organic carbon–carbon or carbon–oxygen backbones found in polyurethanes. Below the glass transition temperature of the matrix, the foam may become brittle, reducing damping performance. At higher temperatures, the matrix may degrade or lose elasticity. Further research into utilizing other high-temperature polymer matrices, such as polyimides or specific fluoropolymers, could potentially extend the thermal stability of MR foams beyond currently known limits, thereby broadening their application scope in demanding thermal environments.

To address the limited thermal stability of MR foams, Zhang et al. [2] proposed increasing the magnetic particle content, particularly iron particles. They investigated the thermal degradation of magnetic PU foams containing 0–75 wt.% iron particles using thermogravimetric analysis (TGA) in both nitrogen and air atmospheres. The differential thermogravimetric curves showed slower weight loss rates for the magnetic foams compared to pure PU foam. The thermal stability at 50% weight loss for MR foams with 50 and 75 wt.% CIPs was 280 °C and 287 °C, respectively. These results are consistent with previous studies [13] reporting thermal stability of 370 °C and 381 °C for 70 and 80 wt.% CIPs, respectively. Additionally, they suggested that during PU degradation, iron particles may act as fillers, restricting PU molecular chain motion upon heating and, thereby, delaying thermal degradation. A two-stage degradation behavior in nitrogen was also observed: the initial weight loss was attributed to the degradation of hard segments (isocyanate/TDI), while the second-stage degradation was associated with soft segments (polyol/PPG). The DTG curves further revealed the complexity of oxygen-involved degradation, including PU oxidation, iron particle oxidation, and potential metal–polymer interactions during oxidative decomposition.

While increasing magnetic particle content improves thermal stability, this method can also promote non-homogeneous mixing of matrix–filler components, leading to particle aggregation in the struts [177]. This compromises the MR foam’s structural integrity by disrupting uniform cell wall formation, which can lead to premature cell collapse during the foaming process. Such collapse disturbs the intended cellular architecture, negatively impacting mechanical compliance, energy absorption, and magneto-mechanical responsiveness. To address this issue, Saiz-Arroyo et al. [62] explored an alternative strategy involving the incorporation of additives within the PU foam matrix. Additives such as silica nanoparticles, acting as a crosslinker or chain extender, can improve thermal resistance by enhancing intermolecular interactions. However, the effectiveness of such additives is highly dependent on matrix chemistry and environmental conditions. Their study, using thermogravimetric analysis, showed that the addition of 9 wt.% silica nanoparticles to low-density polyethylene resulted in a 14 °C increase in thermal decomposition temperature, demonstrating the potential of additive strategies to improve thermal stability while preserving foam morphology.

Rashid et al. [83] also investigated the use of silica as an additive to enhance the thermal stability of MREs. They utilized 30 wt.% CIPs as the primary filler, along with varying contents of silica nanoparticles (0 to 11 wt.%). Their results revealed that temperatures ranging from 25 to 65 °C diminished the interfacial interactions between the filler and the matrix, consequently affecting the properties of the MREs, where both the tensile properties and MR effect decreased with the increasing temperature. This conclusion was supported by FESEM analysis conducted after rheological tests performed at these specified temperatures. However, the presence of silica was able to improve the thermal stability of the MREs by strengthening the interactions between the filler and the matrix, thereby reducing interfacial defects under the influence of temperature. The thermal stabilities of the MREs were characterized using TGA by examining the onset temperature (*T*_onset_), which refers to the beginning of weight loss. The thermal characterization tests showed that the incorporation of silica improved the thermal stability of the MREs, as evidenced by an increase in the *T*_onset_ from 430.3 to 438.5 °C with an increase in silica content from 0 to 11 wt.%.

While increasing the magnetic particle volume fraction and adding thermally stable particles can enhance the thermal stability of MR solids, these improvements often prove insufficient to broaden their range of applications. Therefore, exploring alternative strategies, such as utilizing matrix materials with inherently higher thermal stability, should be considered to achieve this objective.

### 9.2. Matrix/Particle Interface

Weak bonding between the matrix and particles reduces the performance of MR foams. To ensure adequate bonding, magnetic particles may undergo surface treatments before being integrated into the matrix. These treatments aim to introduce functional groups that enhance adhesion between the particles and the matrix. For example, silane coupling agents can form chemical bonds between the particles and the matrix [178].

Zhao et al. [85] improved the interfacial interaction between silicone rubber and CIPs by applying a coating layer. The CIPs underwent surface modification through polydopamine deposition and n-dodecyltrimethoxysilane (DTMS) grafting. Initially, the CIPs were treated with ethanol under ultrasonication for 20 min to remove impurities and activate the surface, followed by three washes with deionized water. Subsequently, the pre-treated particles were added to a 2 g/L dopamine aqueous solution and mechanically stirred for 12 h at room temperature. Then, 6 wt.% of DTMS (based on the mass of the dopamine aqueous solution) was added to the mixture of CIPs and dopamine solution, and the reaction was carried out at 60 °C for 8 h. Finally, the CIPs were separated from the reactor, washed three times with deionized water, and then dried in a vacuum at 60 °C. The results confirmed the successful deposition of a coating layer with a thickness of approximately 30.6 nm, which had little impact on the magnetic properties of the CIPs. Compared with composites containing non-treated CIPs, composites containing surface-modified CIPs showed an enhanced zero-field modulus due to the improved dispersibility of the particles and their interfacial interactions with the silicone rubber matrix. The tensile strength of anisotropic MREs increased by 31.5% after surface modification of the CIPs. The study of magnetic field-induced changes in viscoelastic properties showed that MREs based on treated CIPs exhibited a superior MR effect compared to the samples containing non-treated CIPs. The relative MR effect of isotropic and anisotropic CIP-based MREs was enhanced by 14.2% and 9.0%, respectively, after CIP surface modification.

Although the coating layer proposed by Zhao et al. successfully improved the tensile strength of the studied MRE by enhancing the matrix/CIP interfacial interaction, further research is needed to improve the matrix/magnetic particle interfacial interaction in MR foams.

Beyond silane coupling and dopamine deposition, several advanced surface treatment strategies show promise, such as grafted polymer brushes and plasma surface functionalization. In the grafted polymer brush technique, dense, covalently tethered polymer chains can be grown from the surfaces of magnetic particles via “grafting-from” methods. These brushes enhance steric stabilization, improve particle dispersion within the matrix, and reduce aggregation [179]. In plasma surface functionalization, cold plasma treatments introduce polar functional groups and increase surface roughness without affecting bulk properties, thereby significantly improving adhesion and wettability between the particles and the polymer matrix [180]. Future research should evaluate the feasibility and long-term durability of these coatings under cyclic loading, magnetic field exposure, and environmental stress. Additionally, exploring synergistic effects between surface treatments and other additives—such as nanoparticle crosslinkers or plasticizers—will be critical for systematically enhancing interfacial bonding and mechanical performance in MR foams.

In addition to fabrication and optimization challenges, the long-term reliability of MR foams raises several critical concerns that must be addressed before their widespread adoption in demanding applications. One major risk is the gradual degradation of the magnetic particle–matrix interface under cyclic mechanical loading and repeated magnetic field exposure, which can lead to particle debonding, polymer chain breakage, and a progressive decline in field responsiveness [83]. Thermal aging of the polymer matrix, particularly under elevated or fluctuating temperatures, may result in embrittlement, loss of elasticity, or oxidative degradation [181]. In porous MR foams, environmental exposure to moisture, oxygen, or UV radiation can further accelerate material deterioration by weakening the matrix or oxidizing the particles. Several strategies have been proposed to mitigate these risks. Surface functionalization of magnetic particles (e.g., using silane coupling agents or polymer coatings) has shown promise in improving particle–matrix adhesion and delaying interfacial failure under cyclic loads [83]. The use of thermally stable or UV-resistant matrix polymers, such as silicone or fluorinated elastomers, can reduce susceptibility to thermal aging and environmental degradation. While these methods have demonstrated effectiveness in controlled settings, comprehensive long-term validation under real-world operating conditions is still limited. Therefore, future research should incorporate accelerated aging protocols, fatigue testing, and environmental durability studies to systematically assess the lifecycle performance of MR foams and validate the practicality of current mitigation strategies.

To effectively tackle the persistent challenges in MR foam development, future research should increasingly leverage novel technologies and interdisciplinary strategies. For instance, additive manufacturing and digital light processing (DLP)-based 3D printing (a photopolymerization technique enabling high-resolution fabrication of complex geometries) offer new avenues for fabricating architected MR foam structures with precise control over pore geometry and anisotropy. These methods can help overcome limitations in particle dispersion and structural uniformity. In situ magnetic field-assisted curing, when integrated with real-time imaging or rheological monitoring, can further optimize particle alignment and matrix curing kinetics. On the modeling side, coupling finite element analysis (FEA) with data-driven AI models may enable rapid evaluation of foam behavior across scales and operating conditions, facilitating property prediction and design optimization. Moreover, interdisciplinary collaborations—e.g., materials scientists developing responsive matrices, control engineers embedding sensors and actuators, and data scientists deploying predictive algorithms—will be essential for translating MR foams into next-generation adaptive systems. A clearer understanding of these synergistic pathways will not only help mitigate existing material limitations but also unlock new applications in wearable haptics, vibration isolation in autonomous systems, and lightweight reconfigurable aerospace structures. Ultimately, such advances will position MR foams as key enablers in the development of smart, responsive, and sustainable engineering solutions.

## 10. Conclusions

MR foams have established themselves as a versatile and promising material class, offering solutions to long-standing issues in MR material applications, such as sedimentation and fluid leakage. Their unique combination of porous structures and magnetic responsiveness enables a broad spectrum of applications, from vibration control and energy harvesting to acoustic absorption and aerospace components. Advances in fabrication techniques, particularly the shift toward in situ integration of magnetic particles, have enhanced dispersion, improved mechanical performance, and enabled greater control over anisotropic behavior.

The integration of AI-driven tools, such as artificial neural networks (ANNs), genetic algorithms, and machine learning-based surrogate models, is further accelerating the design and development of MR foams. These tools have been successfully applied to predict viscoelastic properties (e.g., storage and loss moduli) across varying field and frequency ranges, optimize magnetic particle concentration, and guide the selection of processing conditions to tailor foam morphology. AI also enables the inverse design of MR foams with targeted mechanical or magnetic responses by uncovering hidden relationships in complex datasets. Together, these capabilities reduce experimental workload and enhance performance optimization across multiple design objectives.

Despite these advancements, several challenges remain. These include achieving uniform particle distribution, maintaining mechanical and thermal stability under cyclic loading, and navigating trade-offs between conflicting material requirements. For instance, increasing magnetic particle content improves magnetic responsiveness but can shorten the linear viscoelastic (LVE) region, and adding thermally stable fillers can improve thermal resistance but may lead to the formation of particle aggregates when excessive nanoparticles are added, which disrupts foam morphology and weakens overall structural uniformity. To address these issues, promising strategies include surface functionalization of magnetic particles (e.g., silane coupling or polymer coatings) to improve dispersion, the incorporation of multifunctional additives to boost both mechanical and thermal performance, and advanced processing techniques such as constrained foaming or in situ particle alignment to ensure structural uniformity.

Looking ahead, the future of MR foams lies in the development of multifunctional, tunable systems for emerging applications such as soft robotics, adaptive acoustics, and biomedical devices—fields that demand lightweight, reconfigurable, and field-responsive materials. Achieving these goals will require close interdisciplinary collaboration. Materials scientists must develop new matrix formulations with enhanced elasticity, thermal stability, and compatibility with magnetic fillers. Chemists can contribute by designing advanced surface modification techniques to stabilize the particle–matrix interface under operational stresses. Mechanical engineers are needed to refine foam architectures and fabrication processes to control anisotropy, porosity, and energy dissipation. At the same time, AI experts and data scientists will play a pivotal role in modeling structure–property relationships, guiding optimization, and accelerating material discovery through data-driven approaches. The convergence of these efforts will be essential to unlock the full potential of MR foams in next-generation smart and adaptive systems.

## Data Availability

This is a review paper, and all data supporting the findings of this study are included within the manuscript.

## Abbreviations

The following abbreviations are used in this manuscript:

MR	Magnetorheological
AI	Artificial Intelligence
CIP	Carbonyl Iron Particles
PU	Polyurethane
MRE	MR Elastomer
LVE	Linear Viscoelastic
PES	Poly (ether sulfone)
PEN	Poly (ethylene-2,6-naphthalate)
PI	Polyimide
HP-55	Hypromellose Phthalate
PPG	Polyether Polyol
DBTDL	Dibutyltin Dilaurate
MDI	Methylene Diphenyl Diisocyanate
DBP	Dibutyl Phthalate
NH_4_HCO_3_	Ammonium Bicarbonate
CNT	Carbon Nanotubes
RVE	Representative Volume Element
ANN	Artificial Neural Network
GA	Genetic Algorithm
GWO	Grey Wolf Optimization
LSSVM	Least Squares Support Vector Machine
SVR	Support Vector Regression
BP	Backpropagation
ELM	Extreme Learning Machine
MLP	Multilayer Perceptron
LM	Levenberg-Marquardt
RMSE	Root-Mean-Square Error
SLFN	Single Hidden Layer Feedforward Neural Network
MD-CBA	Magnetic Dipole Theory and CNN-BiLSTM-Attention
FFNN	Feedforward Neural Network
ANNOFA	ANN Optimized by a Fuzzy Algorithm
CPVC	Critical Particle Volume Concentration
SAC	Sound Absorption Coefficient
MRTTC	MR materials-based Tactile Transfer Cell
RMIS	Robot-assisted Minimally Invasive Surgery
TGA	Thermogravimetric Analysis
DTMS	Dodecyltrimethoxysilane
